# Reducible problem for a class of almost-periodic non-linear Hamiltonian systems

**DOI:** 10.1186/s13660-018-1787-7

**Published:** 2018-07-28

**Authors:** Muhammad Afzal, Tariq Ismaeel, Muhammad Jamal

**Affiliations:** 1 School of Mathematical Sciences, Ocean University of China, Qingdao, P.R. China; 2 Department of Mathematics, Government College University, Lahore, Pakistan; 3 Department of Mathematics and Statistics, Pir Mehr Ali Shah Arid Agriculture University, Rawalpindi, Pakistan

**Keywords:** Almost-periodic matrix, Reducibility, KAM iteration, Hamiltonian systems, Small divisors

## Abstract

This paper studies the reducibility of almost-periodic Hamiltonian systems with small perturbation near the equilibrium which is described by the following Hamiltonian system:

$$\frac{dx}{dt} = J \bigl[{A} +\varepsilon{Q}(t,\varepsilon) \bigr]x+ \varepsilon g(t,\varepsilon)+h(x,t,\varepsilon). $$

It is proved that, under some non-resonant conditions, non-degeneracy conditions, the suitable hypothesis of analyticity and for the sufficiently small *ε*, the system can be reduced to a constant coefficients system with an equilibrium by means of an almost-periodic symplectic transformation.

## Introduction

In this paper we are studying the reducibility of the following almost-periodic Hamiltonian system:

1 $$\begin{aligned} \frac{dx}{dt} = J \bigl[{A} + \varepsilon{Q}(t,\varepsilon) \bigr]x +\varepsilon g(t,\varepsilon)+h(x,t,\varepsilon),\quad x\in \mathbf{R}^{2N}, \end{aligned}$$

where *J* is an anti-symmetric symplectic matrix, *A* is a $2N \times 2N$ symmetric constant matrix with possible multiple eigenvalues, and $Q(t)$ is an analytic almost-periodic symmetric $2N \times2N$ matrix with respect to *t*, $g(t,\varepsilon)$ and $h(x,t,\varepsilon)$ are almost-periodic 2*N*-dimensional vector-valued functions with respect to *t*, with basic frequencies $\omega=(\omega_{1},\omega_{2},\ldots)$ and $h(x,t)=O(x^{2})$ ($x\rightarrow0$), and

$$J=\left ( \begin{matrix} 0 & I_{N} \\ -I_{N} & 0 \end{matrix} \right ) , $$

where $I_{N}$ is a $N\times N$ identity matrix and *ε* is a sufficiently small parameter. First of all we will recall some previous results in the field of reducibility for analytic differential systems. Consider the differential equation

2 $$\begin{aligned} \frac{dx}{dt} = A(t)x,\quad {x\in\mathbf{R}^{m}}, \end{aligned}$$

where $A(t)$ is an almost-periodic matrix. We call the transformation $x= P(t)y$ almost-periodic Lyapunov–Perron (L-P) transformation, if $P(t)$ is non-singular and *P*, ${P}^{-1}$, and *Ṗ* are almost periodic. The transformed equation is

3 $$\begin{aligned} \frac{dy}{dt} = {C}(t)y, \end{aligned}$$

where ${C}= {P}^{-1}(AP-\dot{P})$. If there exists an almost-periodic L-P transformation such that ${C}(t)$ is a constant matrix, then we call equation (2) reducible.

In recent years, many researchers have devoted themselves to the study of the reducibility of finite dimensional systems by means of the KAM methods. The well-known Floquet theorem states that every periodic differential equation (2) can be reduced to a constant coefficients differential equation (3) by means of a periodic change of variables with the same period as ${A}(t)$. But, if ${A}(t)$ is quasi-periodic (q-p), then there is an example in [1] which illustrates that (2) is irreducible. In 1981, Johnson and Sell [2] showed that if $A(t)$ the quasi-periodic matrix satisfies “full spectrum” conditions, then (2) is reducible. In 1992, Jorba and Simó [3] proved the reducibility result of linear quasi-periodic systems like (5) for the constant matrix *A* with distinct eigenvalues. In 1999, Xu [4] proved the reducibility result of linear quasi-periodic systems like (5) for the constant matrix *A* with multiple eigenvalues. In 1996, Jorba and Simó [5] considered the quasi-periodic system

4 $$ \frac{dx}{dt} = \bigl[ {A}+\varepsilon{Q}(t) \bigr] x+ \varepsilon {g}(t)+ {h}(x,t),\quad {x\in\mathbf{R}^{m},} $$

where the constant matrix *A* has distinct eigenvalues. They proved that system (4) is reducible for $\varepsilon\in E$ using the non-resonant conditions and non-degeneracy conditions, where *E* is the non-empty Cantor subset such that $E\subset(0,\varepsilon_{0})$. Instead of quasi-periodic reduction to a constant coefficient linear systems, in 1996, Xu and You [6] proved the reducibility of the linear almost-periodic differential equation

5 $$ \frac{dx}{dt} = \bigl[ {A}+\varepsilon{Q}(t) \bigr] x,\quad {x \in\mathbf{R}^{m},} $$

where the constant matrix *A* has different eigenvalues and ${Q}(t)$ is an $m \times m$ analytic almost-periodic matrix with frequencies $\omega= (\omega_{1},\omega_{2},\ldots)$. Under some small divisor conditions and for most sufficiently small *ε*, they proved that system (5) is reducible to the constant coefficient system by an affine almost-periodic transformation. In 2013, Qiu and Li [7] considered the following non-linear almost-periodic differential equation:

6 $$ \frac{dx}{dt} = \bigl[ {A}+\varepsilon{a}(t) \bigr] {x}^{2n+1} + {h}(x,t, \varepsilon)+ {f}(x,t,\varepsilon),\quad {x\in \mathbf{R},} $$

where ${n\geq0}$ is an integer, *A* is a positive number, *ε* is a small parameter, *h* is a higher order term, and *f* is a small perturbation term. They proved that under some suitable conditions and using the KAM method system (6) can be reduced to a suitable normal form with zero as an equilibrium point by an affine almost-periodic transformation, so it has an almost-periodic solution near zero.

In 2015, Li et al. [8] considered the following analytic quasi-periodic Hamiltonian system:

7 $$ \frac{dx}{dt} = \bigl[ {A}+\varepsilon{Q}(t) \bigr] x+ \varepsilon {g}(t)+ {h}(x,t),\quad {x\in\mathbf{R}^{2m}}, $$

where the constant matrix *A* has multiple eigenvalues, *Q*, *g*, and *h* are quasi-periodic with respect to *t* and $h={O}(x ^{2})$ ($x\rightarrow0$). They proved that by using the non-resonant conditions, non-degeneracy conditions, and a suitable hypothesis of analyticity, the Hamiltonian system (7) can be changed to another Hamiltonian system with an equilibrium by a q-p symplectic transformation.

In this paper we are going to extend the results of [5] to the almost-periodic Hamiltonian system (1).

This paper is organized as follows. In Sect. 2, statement of the main result is given, in Sect. 3 we give some lemmas which are essential for the proof of the main result, in Sect. 4 the first KAM step is given, in Sect. 5 the main result is proved, and finally, in Sect. 6 conclusion of the paper is given.

### Definitions and notations

To state our main result, we need some definitions and notations.

#### Definition 1.1

We say that a function *f* is a quasi-periodic function of time *t* with basic frequencies $\omega=(\omega_{1},\omega_{2},\ldots,\omega_{d})$ if $f(t)=F(\theta_{1},\theta_{2},\ldots,\theta_{d})$, where *F* is 2*π* periodic in all its arguments $\theta_{j}=\omega_{j} t$ for $j = 1,2,\ldots,d$. *f* will be called analytic quasi-periodic in a strip of width *ρ* if *F* is analytic on $D_{\rho}=\{\theta|| \Im\theta_{j}|\leq\rho, j=1,2,\ldots,d\}$. In this case we denote the norm by $\|f\|_{\rho}=\sum_{k\in\mathbf{Z}^{d}}|F_{k}|e^{\rho|k|}$. A function *f* is almost-periodic if $f(t)=\sum_{n=1}^{\infty}f_{n}(t)$, where $f_{n}(t)$ are all quasi-periodic for $n=1,2,\ldots$ .

#### Definition 1.2

Suppose that $A(t)= (a_{sj}(t))$ is a quasi-periodic $m \times m$ matrix. If every $a_{sj}(t)$ is analytic on $D_{\rho}$, then we call $A(t)$ analytic on $D_{\rho}$.The norm of $A(t)$ is defined as follows:

$$\begin{aligned} \bigl\Vert A(t)\bigr\Vert _{\rho}=m \times\max_{1\leq s,j \leq r} \bigl\Vert a_{sj}(t)\bigr\Vert _{ \rho}. \end{aligned}$$

If *A* is a constant matrix, the norm of *A* is defined as follows:

$$\begin{aligned} \Vert A \Vert =m \times\max_{1\leq s,j \leq r} \vert a_{sj} \vert . \end{aligned}$$

Write $B(0,b)= \{ x\in\mathbf{C}| |x|\leq b\}$ and $\Delta_{b, \rho}= B(0,b) \times D_{\rho}$.

#### Definition 1.3

Let $h(x,t)$ be real analytic in *x* and *t* on $\Delta_{b,\rho}$, and let $h(x,t)$ be quasi-periodic with respect to *t* with frequency *ω*. Then $h(x,t)$ can be expanded as a Fourier series as follows:

$$\begin{aligned} h(x,t) =\sum_{k\in\mathbf{Z}^{d}} h_{k}(x) e^{i\langle k,\theta \rangle}. \end{aligned}$$

Then

$$\begin{aligned} \Vert h\Vert _{\Delta_{b,\rho}} =\sum_{k\in\mathbf{Z}^{d}} \vert h_{k} \vert _{b} e ^{\rho \vert k\vert }, \end{aligned}$$

where $h_{k}(x)= \sum^{\infty}_{n=0} h_{nk} x^{n}$ and $|h_{k}|_{b}= \sup_{x\in B(0,b)}\sum^{\infty}_{n=0} |h_{nk}| |x|^{n}$. It is easy to see that

$$\begin{aligned} \Vert h_{1} h_{2}\Vert _{\Delta_{b,\rho}} \leq \Vert h_{1}\Vert _{\Delta_{b,\rho}} \Vert h_{2}\Vert _{\Delta_{b,\rho}}. \end{aligned}$$

The aim of the study is to develop the reducibility for the almost-periodic non-linear Hamiltonian system (1). To take over the difficulty from the infinite frequency which generates the small divisors problem, we need a stronger norm. Inspired by the works of [4, 5], and [8], in this paper, we allow *Q*, *g*, and *h* to be the classes of almost-periodic matrices. Our study is about the reducibility of almost-periodic Hamiltonian systems to [4] and [8]. So, the usual LP transformation for KAM iteration should not only be almost-periodic but also symplectic, which preserves the Hamiltonian structure. For this purpose, let us introduce “spatial structure”, “approximation function”, and some related definitions.

#### Definition 1.4

([9])

Let *τ* be a set of some subsets of the natural number set **N**. Then $(\tau,[\cdot])$ is called a finite spatial structure in **N** if *τ* satisfies:

1. $\emptyset\in\tau$;
2. If $\Lambda_{1}, \Lambda_{2}\in\tau$, then $\Lambda_{1}\cup\Lambda _{2}\in\tau$;
3. $\bigcup_{\Lambda\in\tau}\Lambda=\mathbf{N}$,

and $[\cdot]$ is a weight function defined on *τ* such that $[\emptyset]=0$, $[\Lambda_{1}\cup\Lambda_{2}]\leq[\Lambda_{1}]+[ \Lambda_{2}]$.

Denote the weight value by $[k]= \inf_{\operatorname{supp}k\subset\Lambda,\Lambda\in\tau}[\Lambda]$. Write $|k|=\sum_{s=1}^{\infty}|k_{s}|$.

#### Definition 1.5

([4])

If ${U}(t)=\sum_{\Lambda\in\tau}{U}_{\Lambda}(t)$, where ${U}_{\Lambda}(t)$ are quasi-periodic matrices with basic frequencies $\omega_{\Lambda}=\{\omega_{s}|s\in\Lambda\}$, then ${U}(t)$ is known as an almost-periodic matrix with spatial structure $(\tau,[\cdot])$ and basic frequencies *ω*, which is the maximum subset of $\cup\omega_{\Lambda}$ in the sense of integer modular. Denote the average of ${U}(t)$ by *U̅*, where

$$\overline{U}=\lim_{T\rightarrow\infty}\frac{1}{2T} \int_{-T}^{T}U(t)\,dt. $$

Let ${U}(t)=\sum_{\Lambda\in\tau}{U}_{\Lambda}(t)$, for $z>0$, $\rho>0$,

$$\begin{aligned} \big|\!\big|\!\big|{U}(t)\big|\!\big|\!\big|_{z,\rho}=\sum_{\Lambda\in\tau}e^{z[\Lambda]} \bigl\Vert {U} _{\Lambda}(t)\bigr\Vert _{\rho} \end{aligned}$$

is called a weight norm with finite spatial structure $(\tau,[\cdot ])$.

#### Definition 1.6

([10])

Δ is called an approximation function if

1. $\Delta:[0,\infty)\rightarrow[1,\infty)$ is an increasing function and it satisfies $\Delta(0)=1$;
2. $\frac{\log\Delta(t)}{t}$ is decreasing on $[0,\infty)$;
3. $\int_{0}^{\infty}\frac{\log\Delta(t)}{t^{2}}\,dt<\infty$.

#### Remark

If Δ is an approximation function, then so is $\Delta^{4}$.

#### Definition 1.7

Let $h(x,t)= \sum_{\Lambda\in\tau}{h}_{\Lambda}(x,t)$ with frequency $\omega=(\omega_{1}, \omega_{2}, \ldots)$ and with finite spatial structure $(\tau,[\cdot])$, for $z>0$, $\rho>0$,

$$\begin{aligned} \big|\!\big|\!\big|{h}(x,t)\big|\!\big|\!\big|_{z,\Delta_{b,\rho}}=\sum_{\Lambda\in\tau}e^{z[ \Lambda]} \bigl\Vert {h}_{\Lambda}(x,t)\bigr\Vert _{\Delta_{b,\rho}} \end{aligned}$$

is known as the weight norm of $h(x,t)$.

#### Definition 1.8

A matrix *S* is said to be symplectic if $SJS^{T}=J$, where $S^{T}$ represents the transpose of *S* and $J= ( {\scriptsize\begin{matrix}{} 0 & I_{N} \cr -I_{N} & 0 \end{matrix}} ) $, where $I_{N}$ is an $N \times N$ identity matrix.

For a map $\psi(t,x):\mathbf{R}^{2N}\rightarrow\mathbf{R}^{2N}$, let $\frac{\partial\psi}{\partial x}$ denote the Jacobian of *ψ* with respect to *x*, that is, $\frac{\partial\psi}{\partial x}= ( \frac{ \partial\psi_{i}}{\partial x_{j}} ) _{2N\times2N}$.

#### Definition 1.9

$\psi(t,x)$ is symplectic if and only if the Jacobian of *ψ* with respect to *x* is symplectic, i.e., $\frac{\partial\psi}{\partial x} J {\frac{\partial\psi}{\partial x}} ^{T}= J$.

#### Definition 1.10

A matrix *A* is said to be Hamiltonian if and only if $A=JB$, where *B* is a symmetric matrix and *J* is defined as above.

For our problem, the non-resonant conditions will be

$$\bigl\vert \lambda_{s}-\lambda_{j}-\sqrt{-1}\langle k, \omega\rangle \bigr\vert \geq\frac{ \alpha}{\Delta^{4}(\vert k\vert )\Delta^{4}([k])} $$

for all $1\leq s\neq j\leq2N$ and $k\in\mathbf{Z}^{\mathbf{N}} \backslash\{0\}$, where $\lambda_{1},\lambda_{2},\ldots,\lambda_{2N}$ are the eigenvalues of *JA*, *ω* is the basic frequencies of ${Q}(t)$, $\Delta(t)$ is an approximation function satisfying $\sum_{k\in\mathbf{Z}^{\mathbf{N}}} \frac{1}{\Delta(|k|)\Delta([k])}<\infty$ and *α* is a small positive constant. From [4] and [9], we can choose the weight function

$$\begin{aligned}{} [\Lambda]=1+\sum_{s\in\Lambda}\log^{p} \bigl(1+ \vert s\vert \bigr), \quad p>2. \end{aligned}$$

## Statement of the main result

### Theorem 2.1

*Suppose that the Hamiltonian system* (1) *in which* *JA* *is a Hamiltonian matrix with possible multiple eigenvalues*, ${Q}(t)= \sum{Q}_{\Lambda }(t)$ *and* ${g}(t)= \sum{g}_{\Lambda}(t)$ *are analytic almost*-*periodic matrices with respect to* *t* *on* $D_{\rho}$, *and* ${h}(x,t)= \sum {h}_{\Lambda}(x,t)$ *is analytic almost*-*periodic matrix with respect to* *t* *and* *x* *on* $\Delta_{b,\rho}$ *with basic frequencies* $\omega=( \omega_{1}, \omega_{2}, \ldots)$ *and has the spatial structure* $(\tau,[\cdot])$ *which depends continuously on the small parameter* *ε*. *Suppose that* *JA* *is a* $2N \times2N$ *matrix with possible multiple eigenvalues* $\lambda_{1},\lambda_{2},\ldots, \lambda_{2N}$ *that can be diagonalized and the multiplicity of the eigenvalues* $\lambda_{s'}$ *is* $r_{s'}$, $s'=1,2,\ldots, l$, $r_{1}+r_{2}+ \cdots+ r_{l}=2N$, $\lambda_{s'}\neq0$, *and* $\lambda_{s'}\neq\lambda_{j'}$ *with* $s'\neq j'$ *and* $1\leq s',j' \leq l$. *Moreover*, *assume that* $h(x,t,\varepsilon)$ *is analytic with respect to* *x* *on the closed ball* $B_{b}(0)$, $h(0,t,\varepsilon)=0$, *and* $D_{x} h(0,t,\varepsilon)=0$ *and* $\varepsilon\in(0,\varepsilon _{0})$ *is a parameter*. *If*

1. *There exists* $z > 0$ *such that* $|\!|\!|{Q}(t)|\!|\!|_{z,\rho}<\infty$, $|\!|\!|{g}(t)|\!|\!|_{z,\rho}<\infty$;
2. (*Non*-*resonant conditions*) $\lambda=(\lambda_{1},\lambda_{2},\ldots, \lambda_{2N})$ *and* $\omega=(\omega_{1}, \omega_{2}, \ldots)$ *satisfy*
  8 $$\begin{aligned}& \bigl\vert \lambda_{s}-\sqrt{-1}\langle k,\omega \rangle \bigr\vert \geq\frac{\alpha _{0}}{\Delta(\vert k\vert )\Delta([k])}, \end{aligned}$$
  9 $$\begin{aligned}& \bigl\vert \lambda_{s}-\lambda_{j}- \sqrt{-1}\langle k,\omega\rangle \bigr\vert \geq\frac{ \alpha_{0}}{\Delta(\vert k\vert )\Delta([k])} \end{aligned}$$
  *for all* $1\leq s,j \leq2N$, $k\in\mathbf{Z}^{\mathbf{N}}\backslash \{0\}$, $\alpha_{0}>0$ *is a small constant and* $\Delta(t)$ *is an approximation function*;
3. (*Non*-*degeneracy conditions*) *Suppose that the unique solution of* $\dot{x}= JAx+\varepsilon g(t)$ *is denoted by* $\underline{x}$. *Assume that* $J({A}+ \varepsilon\overline{Q}^{*})$ *has* 2*N* *different eigenvalues* $\lambda^{1}_{s}(\varepsilon)$ ($1\leq s \leq{2N}$) *which satisfy* $|\lambda^{1}_{k_{1}}(\varepsilon)-\lambda^{1}_{k_{2}}( \varepsilon)|\geq2\eta\varepsilon>0$, $\eta_{2} \geq|\frac{d( \lambda^{1}_{k}(\varepsilon))}{dt}|\geq\eta_{1}>0$, *and* $\eta_{2} \geq|\frac{d(\lambda^{1}_{k_{1}}(\varepsilon)-\lambda^{1}_{k_{2}}( \varepsilon))}{dt}|\geq\eta_{1}>0$, *where* $k, k_{1}, k_{2}=1,2, \ldots, 2N$, $k_{1}\neq k_{2}$, $\varepsilon\in(0,\varepsilon_{0})$, *and* *η*, $\eta_{1}$, *and* $\eta_{2}$ *are positive constants*. *Let* $Q^{*}(t)= Q(t) + \frac{1}{\varepsilon} D_{x}h(\underline{x},t)$ *and* $\overline{Q}^{*}$ *be the average of* $Q^{*}(t)$.
4. $|\!|\!|D_{xx}h(x,t,\varepsilon)|\!|\!|_{z,\Delta_{b,\rho}}\leq K$, *where* $\varepsilon\in(0,\varepsilon_{0})$ *and* $x \in B_{b}(0)$.

Then there exists a Cantor subset $E\subset(0,\varepsilon_{0})$ with positive Lebesgue measure such that the Hamiltonian system (1) is reducible for $\varepsilon\in E$, i.e., there exists an almost-periodic symplectic transformation $x=\psi(t,\varepsilon) y +\varphi(t, \varepsilon)$, where $\psi(t,\varepsilon)$ and $\varphi(t,\varepsilon )$ are almost-periodic with basic frequencies and spatial structure $(\tau, [\cdot])$ as ${Q}(t)$, which transforms (1) into the Hamiltonian system

10 $$\begin{aligned} \frac{dy}{dt}= A_{*}(\varepsilon)+ h_{*}(y,t,\varepsilon), \end{aligned}$$

where $A_{*}(\varepsilon)$ is the constant matrix and $h_{*}(y,t, \varepsilon)$ is of order two in *y*. Furthermore, for small enough $\varepsilon_{0}$, the relative measure of *E* in $(0, \varepsilon _{0})$ is close to 1.

### Remark

In general, we suppose that $g(t)$, $Q(t)$, and $h(x,t)$ depend on *ε*, but for simplicity, in the following we do not represent this dependence.

## Some lemmas

In this section, we will give some results in the form of lemmas which are useful for the proof of Theorem 2.1.

### Lemma 3.1

([4])

*Suppose that* *U* *and* *R* *are almost*-*periodic matrices*, *and they have the same spatial structure and the same frequencies*. *If* $|\!|\!|{U}|\!|\!|_{z,\rho} < +\infty$, $|\!|\!|{R}|\!|\!|_{z,\rho} < +\infty$, *then* *UR* *is an almost*-*periodic matrix and has the same spatial structure and the same frequencies with* *U* *and* *R*, *and*

$$\begin{aligned} |\!|\!|{UR}|\!|\!|_{z,\rho} \leq |\!|\!|{U}|\!|\!|_{z,\rho} |\!|\!|{R} |\!|\!|_{z,\rho}. \end{aligned}$$

### Lemma 3.2

([4])

*Suppose that an analytic almost*-*periodic matrix* ${U}(t)$ *has the spatial structure* $(\tau, [\cdot])$, *and for* $z>0$, $\rho>0$, $|\!|\!|{U}|\!|\!|_{z,\rho} < + \infty$. *Then*, *for the average of* ${U}(t)$, *we have* $\Vert \overline{{U}}\Vert \leq |\!|\!|{U}|\!|\!|_{z, \rho}$.

### Lemma 3.3

([5])

*Let* $\lambda ^{1}_{1},\lambda^{1}_{2}, \ldots, \lambda^{1}_{2l}$ *be the eigenvalues of* $B_{1}$ *satisfying* $|\lambda ^{1}_{s}|>2\nu$, $|\lambda^{1}_{s}-\lambda^{1}_{j}|>2\nu$, *and* $\nu>0$ *with* $s \neq j$. *Consider* $B_{0}$ *to be a matrix such that* $B^{-1}_{0}B_{1}B_{0}= \operatorname{diag}(\lambda^{1}_{1},\ldots,\lambda^{1}_{2l})$. *Define* $\beta_{0}=\max\{\Vert B_{0}\Vert , \Vert B^{-1}_{0}\Vert \}$. *Let* *ς* *be a value such that* $0 < \varsigma< \frac{2\nu}{(6l-1)\beta^{2} _{0}}$. *Then*, *if* *JA* *verifies* $\Vert JA-B_{1}\Vert < \varsigma$, *the following results hold*:

1. *If* $\lambda_{1},\lambda_{2}, \ldots, \lambda_{2l}$ *are the eigenvalues of* *JA*, *we have* $|\lambda_{s}|>\nu$, $|\lambda_{s}-\lambda_{j}|> \nu$, $s\neq j$.
2. *There exists a non*-*singular matrix* *B* *such that* $B^{-1}{JA}B= \operatorname{diag}( \lambda_{1},\lambda_{2}, \ldots, \lambda_{2l})$, *which satisfies* $\Vert B\Vert , \Vert B^{-1}\Vert \leq\beta$, *where* $\beta=2\beta_{0}$.

### Remark

Indeed, by Gerschgorin’s lemma, the result 1 of Lemma 3.3 can be obtained. In our case, if the constant matrix $A_{0}$ can be diagonalized, then the eigenvalues $\lambda^{0}_{s}$ of $A_{0}$ satisfy $|\lambda^{0}_{s}|\geq2\eta_{3}>0$ with a constant $\eta_{3}$, ∀*s*, and $A_{n} - A_{0}=O(\varepsilon)$, where $n \geq1$ represents the *n*th KAM step. So, by Gerschgorin’s lemma, we have that, for small enough *ε*, the eigenvalues $\lambda^{n} _{s}$ of $A_{n}$ satisfy $|\lambda^{n}_{s}|\geq\eta_{3}>0$, ∀*s*. In this article, we denote $\nu= \eta\varepsilon$ and $B_{1} = A_{1}$. Then Lemma 3.3 holds and, moreover, in this article, $\beta_{0}$ is a bounded and positive constant.

### Lemma 3.4

*Consider the system*

11 $$\begin{aligned} \dot{x}= JAx + \varepsilon g(t),\quad x \in\mathbf{R}^{2N}, \end{aligned}$$

*where* $JA \in B_{\varsigma}(A_{1})$ *and* *ς* *is given by Lemma *3.3. *Let the eigenvalues of* *JA* *be* $\lambda_{s}$, *where* $|\lambda _{s}|\geq\eta_{3}>0$ *with a constant* $\eta_{3}$, ∀*s*. *Moreover*, $g(t)=\sum g_{\Lambda}(t)$ *is analytic almost*-*periodic on* $D_{\rho}$ *with frequencies* $\omega=(\omega_{1},\omega_{2},\ldots)$ *and has the spatial structure* $(\tau,[\cdot])$. *Suppose*

12 $$\begin{aligned} \bigl\vert \lambda_{s}-\sqrt{-1}\langle k,\omega \rangle \bigr\vert \geq\frac{\alpha}{ \Delta^{4}(\vert k\vert )\Delta^{4}([k])} \end{aligned}$$

$\forall k \in\mathbf{Z}^{\mathbf{N}}\backslash\{0\}$, *a constant* $\alpha>0$ *and an approximation function* $\Delta(t)$. *Let* $0<\overline{\rho}<\rho$, $0<\overline{z}<z$. *So*, *for equation* (11), *a unique analytic almost*-*periodic solution* $x(t)$ *exists with the same spatial structure and the same frequency as* $g(t)$ *which satisfies* $|\!|\!|x|\!|\!|_{z-\overline{z},\rho-\overline{\rho}}\leq c \varepsilon\frac{ \Gamma(\overline{z})\Gamma(\overline{\rho})}{\alpha} |\!|\!|{g}|\!|\!|_{z, \rho}$, *where* $\Gamma(\rho)= \sup_{t\geq0}[\Delta^{4}(t) e^{- \rho t}$ *and a constant* $c>0$.

### Proof

Making the change of variables $x=By $ and by defining $h(t) = B^{-1} g(t)$, system (11) can be written as

13 $$\begin{aligned} \dot{y}= Dy+\varepsilon h(t), \quad y \in\mathbf{R}^{2N}, \end{aligned}$$

where $D= B^{-1}(JA)B= \operatorname{diag}(\lambda_{1},\lambda_{2}, \ldots, \lambda _{2N})$. Let

$$\begin{aligned}& y_{\Lambda}= \bigl(y^{sj}_{\Lambda} \bigr), \qquad \bigl(y^{sj}_{\Lambda k} \bigr)= \sum_{\operatorname{supp}k\subset\Lambda}y^{sj}_{\Lambda k}e^{\sqrt{-1} \langle k,\theta\rangle}, \\& h_{\Lambda}= \bigl(h^{sj}_{\Lambda} \bigr),\qquad \bigl(h^{sj}_{\Lambda k} \bigr)= \sum_{\operatorname{supp}k\subset\Lambda}h^{sj}_{\Lambda k}e^{\sqrt{-1} \langle k,\theta\rangle}, \end{aligned}$$

where, ${\theta}=\omega t$.

Substitute these into $\dot{y}_{\Lambda}= Dy_{\Lambda}+\varepsilon {h}_{\Lambda}$, and by equating the coefficients on both sides, we obtain

$$y^{sj}_{\Lambda k}= \varepsilon\frac{h^{sj}_{\Lambda k}}{\lambda_{s}- \sqrt{-1}\langle k,\omega\rangle}. $$

So, by equation (12), we get

$$\begin{aligned} \bigl\Vert y^{sj}_{\Lambda}\bigr\Vert _{\rho-\overline{\rho}} \leq & \varepsilon \biggl(\frac{1}{ \eta_{3}}+\sum_{\operatorname{supp}k\subset\Lambda} \frac{\Delta^{4}(\vert k\vert )e ^{-\overline{\rho} \vert k\vert }}{\alpha}\Delta^{4} \bigl([k] \bigr) \biggr) \bigl\vert h^{sj}_{\Lambda k} \bigr\vert e ^{\rho \vert k\vert } \\ \leq & c \varepsilon\frac{\Gamma(\overline{\rho})\Delta^{4}([ \Lambda])}{\alpha}\bigl\Vert h^{sj}_{\Lambda} \bigr\Vert _{\rho}, \end{aligned}$$

where $c>0$ is a constant. Thus

$$\Vert y_{\Lambda} \Vert _{\rho-\overline{\rho}}\leq c \varepsilon \frac{ \Gamma(\overline{\rho})\Delta^{4}([\Lambda])}{\alpha} \Vert h_{\Lambda }\Vert _{\rho}. $$

Let $y =\sum_{\Lambda\in\tau} y_{\Lambda}$. From Definition 1.2, we have

$$\begin{aligned} |\!|\!|y|\!|\!|_{z-\overline{z},\rho-\overline{\rho}} =& \sum_{\Lambda\in\tau} \Vert y_{\Lambda} \Vert _{\rho-\overline{\rho}}e ^{(z-\overline{z})[\Lambda]} \\ \leq& c \varepsilon\sum_{\Lambda\in\tau}\frac{\Gamma(\overline{ \rho})\Delta^{4}([\Lambda])}{\alpha} \Vert {h}_{\Lambda} \Vert _{\rho}e ^{z[\Lambda]-\overline{z}[\Lambda]} \\ \leq& c \varepsilon\frac{\Gamma(\overline{\rho})\Gamma( \overline{z})}{\alpha} |\!|\!|{h}|\!|\!|_{z,\rho}. \end{aligned}$$

By Remark of Lemma 3.3, we have $\Vert B\Vert .\Vert B^{-1}\Vert \leq c_{0}$, where $c_{0}>0$ is a constant. Then, since $|\!|\!|h|\!|\!|_{z,\rho}\leq \Vert B^{-1}\Vert |\!|\!|g|\!|\!|_{z,\rho}$ and $|\!|\!|x|\!|\!|_{z,\rho}\leq \Vert B\Vert |\!|\!|y|\!|\!|_{z,\rho}$, we have

$$\begin{aligned} |\!|\!|x|\!|\!|_{z-\overline{z},\rho-\overline{\rho}}\leq c \varepsilon\frac{ \Gamma(\overline{z})\Gamma(\overline{\rho})}{\alpha} |\!|\!|{g} |\!|\!|_{z, \rho}. \end{aligned}$$

 □

### Lemma 3.5

*Let* $h: B_{b}(0) \subset\mathbf{R}^{l} \rightarrow \mathbf{R}^{l}$ *be a* $C^{2}$ *function that satisfies* $h(0)=0$, $\frac{d(h(0))}{dx}=0$, $\Vert \frac{d^{2}(h(x))}{dx^{2}}\Vert \leq K$, $\forall x \in B_{b}(0)$, *where* $B_{b}(0)$ *is a ball centered at* 0 *and having radius* *b*, *where* *K* *is a constant*. *Then* $\Vert h(x)\Vert \leq\frac{K}{2}\Vert x\Vert ^{2}$, $\Vert \frac{d(h(x))}{dx}\Vert \leq K\Vert x\Vert $.

For the proof of Lemma 3.5, see [5].

### Lemma 3.6

*Let*

14 $$\begin{aligned} \dot{P} = JAP - PJA + Q, \end{aligned}$$

*where* *JA* *is a* $2N \times2N$ *Hamiltonian matrix and* $JA \in B_{ \varsigma}(A_{1})$, *the eigenvalues of* $A_{1}$ *are* $\lambda^{1}_{1}, \lambda^{1}_{2}, \ldots,\lambda^{1}_{2N}$ *with* $|\lambda^{1}_{s}|>2 \eta\varepsilon$ *and* $|\lambda^{1}_{s}-\lambda^{1}_{j}|>2\eta \varepsilon$ *for* $s \neq j$, *and* *ς* *can be found in Lemma *3.3. *Let* $\lambda_{s}$, $1\leq s \leq2N$, *with* $\lambda_{s}\neq0$ *be the eigenvalues of* *JA*. *Moreover*, $Q(t) = \sum_{\Lambda\in\tau} Q _{\Lambda}(t)$ *is an analytic almost*-*periodic Hamiltonian matrix on* $D_{\rho}$ *with frequencies* $\omega=(\omega_{1},\omega_{2}, \ldots)$ *and with finite spatial structure* $(\tau,[\cdot])$. $\overline{Q}=0$, *where* *Q̅* *is the average of* $Q(t)$. *Let*

15 $$\begin{aligned} \bigl\vert \lambda_{s} - \lambda_{j} - \sqrt{-1}\langle k,\omega\rangle \bigr\vert \geq\frac{\alpha}{\Delta^{4}(\vert k\vert )\Delta^{4}([k])}, \end{aligned}$$

$\forall k \in\mathbf{Z}^{\mathbf{N}}\backslash\{0\}$, $\alpha>0$ *is a constant and* $\Delta(t)$ *is an approximation function*, *and* $|\lambda_{k_{1}}-\lambda_{k_{2}}|\geq\eta\varepsilon$, $k_{1} \neq k_{2}$. *Let* $0<\overline{\rho}<\rho$, $0<\overline{z}<z$. *Then we have a unique analytic almost*-*periodic Hamiltonian matrix* $P(t)$ *with the same spatial structure and the same frequencies as* $Q(t)$, *which solves equation* (14) *and satisfies*

$$\begin{aligned} |\!|\!|P|\!|\!|_{z-\overline{z},\rho-\overline{\rho}}\leq c \frac{\Gamma( \overline{z})\Gamma(\overline{\rho})}{\alpha} |\!|\!|{Q}|\!|\!|_{z,\rho} , \end{aligned}$$

*where* $\Gamma(\rho)= \sup_{t\geq0}[\Delta^{4}(t) e^{-\rho t}$ *and* $c>0$ *is a constant*.

### Proof

We can suppose that the matrix *B* is as in Lemma 3.4. Making the setting $P=BVB^{-1}$ and $R=B^{-1}QB$, equation (14) can be written as

$$\begin{aligned} \dot{V} = DV - VD + R, \end{aligned}$$

where $D=\operatorname{diag}(\lambda_{1},\lambda_{2},\ldots,\lambda_{2N})$. Let

$$\begin{aligned}& R_{\Lambda}= \bigl(r^{sj}_{\Lambda} \bigr), \qquad \bigl(r^{sj}_{\Lambda k} \bigr)= \sum_{\operatorname{supp}k\subset\Lambda}r^{sj}_{\Lambda k}e^{\sqrt{-1} \langle k,\theta\rangle}, \\& V_{\Lambda}= \bigl(v^{sj}_{\Lambda} \bigr), \qquad \bigl(v^{sj}_{\Lambda k} \bigr)= \sum_{\operatorname{supp}k\subset\Lambda}v^{sj}_{\Lambda k}e^{\sqrt{-1} \langle k,\theta\rangle}, \end{aligned}$$

where ${\theta}=\omega t$. Substitute these into $\dot{V}_{\Lambda}= D V_{\Lambda}- V_{\Lambda}D + R_{\Lambda}$, and by equating the coefficients on both sides, we have $v^{sj}_{\Lambda0}=0$; or

$$v^{sj}_{\Lambda k}=\frac{r^{sj}_{\Lambda k}}{\lambda_{s}-\lambda_{j}- \sqrt{-1}\langle k,\omega\rangle}\quad \mbox{for } k\neq0. $$

As *Q* is analytic on $D_{\rho}$, therefore $R=B^{-1}QB$ is also analytic on $D_{\rho}$. So, by using equation (15), we have

$$\begin{aligned} \bigl\Vert v^{sj}_{\Lambda}\bigr\Vert _{\rho-\overline{\rho}} \leq & \sum_{\operatorname{supp}k\subset\Lambda}\frac{\Delta^{4}(\vert k\vert )e^{-\overline{ \rho} \vert k\vert }}{\alpha}\Delta^{4} \bigl([k] \bigr) \bigl\vert r^{sj}_{\Lambda k} \bigr\vert e^{\rho \vert k\vert } \\ \leq& \frac{\Gamma(\overline{\rho})\Delta^{4}([\Lambda])}{ \alpha}\bigl\Vert r^{sj}_{\Lambda k}\bigr\Vert _{\rho}. \end{aligned}$$

Thus

$$\Vert P_{\Lambda} \Vert _{\rho-\overline{\rho}}\leq\frac{\Gamma(\overline{ \rho})\Delta^{4}([\Lambda])}{\alpha} \Vert R_{\Lambda} \Vert _{\rho}. $$

Let $V=\sum_{\Lambda\in\tau}{V}_{\Lambda}$. From Definition 1.4, we have

$$\begin{aligned} |\!|\!|V |\!|\!|_{z-\overline{z},\rho-\overline{\rho}} =& \sum_{\Lambda\in\tau} \Vert {V}_{\Lambda} \Vert _{\rho-\overline{\rho}}e ^{(z-\overline{z})[\Lambda]} \\ \leq&\sum_{\Lambda\in\tau}\frac{\Gamma(\overline{\rho})\Delta ^{4}([\Lambda])}{\alpha} \Vert {R}_{\Lambda} \Vert _{\rho}e^{z[\Lambda]- \overline{z}[\Lambda]} \\ \leq&\frac{\Gamma(\overline{\rho})\Gamma(\overline{z})}{\alpha }|\!|\!|{R}|\!|\!|_{z,\rho}. \end{aligned}$$

By Remark of Lemma 3.3, we have $\Vert B\Vert .\Vert B^{-1}\Vert \leq c_{0}$, where $c_{0}>0$ is a constant. Then, by using Lemmas 3.1 and 3.2, we can write

$$\begin{aligned} |\!|\!|P |\!|\!|_{z-\overline{z},\rho-\overline{\rho}}\leq \Vert B\Vert |\!|\!|V |\!|\!|_{z- \overline{z},\rho-\overline{\rho}}\bigl\Vert B^{-1}\bigr\Vert \end{aligned}$$

and

$$\begin{aligned} |\!|\!|R |\!|\!|_{z,\rho}\leq\bigl\Vert B^{-1}\bigr\Vert |\!|\!|Q |\!|\!|_{z,\rho} \Vert B\Vert . \end{aligned}$$

Thus,

$$\begin{aligned} |\!|\!|P|\!|\!|_{z-\overline{z},\rho-\overline{\rho}}\leq c \frac{\Gamma( \overline{z})\Gamma(\overline{\rho})}{\alpha} |\!|\!|{Q}|\!|\!|_{z,\rho}. \end{aligned}$$

Now we prove that $P=\sum_{\Lambda\in\tau} P_{\Lambda}$ is Hamiltonian. To prove *P* is Hamiltonian, we need to prove that $P_{J}$ is symmetric in $P_{J}= J^{-1}P$. As *JA* and $Q = \sum_{\Lambda\in\tau} Q_{\Lambda}$ are Hamiltonian, then by definition, we can write $Q= J Q_{J}$, where *A* and $Q_{J}$ are symmetric. Below we prove that $P_{J}$ is symmetric. Substituting $P= JP_{J}$ and $Q= J Q_{J}$ into equation (14), we have

16 $$ \dot{P}_{J} = AJ P_{J} - P_{J} JA + Q_{J}, $$

and transposing equation (16), we have

$$\begin{aligned} \dot{P}^{T}_{J} = AJ P^{T}_{J} - P^{T}_{J} JA + Q_{J}. \end{aligned}$$

It is easy to see that $J P_{J}$ and $J P^{T}_{J}$ are solutions of equation (14); moreover, $\overline{JP_{J}}=\overline{JP^{T}_{J}}=0$. Since the solution of equation (14) is unique with $\overline{P}=0$, we have that $JP_{J}=JP^{T}_{J}$, which proves that *P* is Hamiltonian. □

### Lemma 3.7

*Consider the Hamiltonian system*

17 $$\begin{aligned} \frac{dx}{dt} = J \bigl[{A} + \varepsilon{Q}(t) \bigr]x + \varepsilon g(t) + h(x,t), \end{aligned}$$

*where* *JA* *is a Hamiltonian matrix of dimension* $2N \times2N$, $JA \in B_{\varsigma}(A_{1})$ *with* *ς* *being given by Lemma *3.3, *and* $\lambda_{s}$ *are eigenvalues of* *JA* *with* $|\lambda_{s}| \geq\eta_{3}>0$, $\forall1\leq s \leq2N$. *Suppose that* $Q(t) = \sum_{\Lambda\in\tau}Q_{\Lambda}(t)$, $g(t) = \sum_{\Lambda\in\tau}g_{\Lambda}(t)$ *are analytic almost*-*periodic on* $D_{\rho}$, *and* $h(x,t) = \sum_{\Lambda\in\tau}h_{\Lambda}(x,t)$ *is an almost*-*periodic analytic matrix with respect to* *t* *and* *x* *on* $\Delta_{b,\rho}$ *with frequencies* $\omega=(\omega_{1},\omega_{2}, \ldots)$ *and has a finite spatial structure* $(\tau,[\cdot])$. *Suppose also that* $h(x,t)$ *is analytic with respect to* *x* *on* $B_{b}(0)$ *and satisfies* $\Vert D_{xx}h(x,t, \varepsilon)\Vert \leq K$, $\forall x \in B_{b}(0)$. *Moreover*,

$$\begin{aligned} \bigl\vert \lambda_{s}-\sqrt{-1}\langle k,\omega\rangle \bigr\vert \geq\frac{\alpha}{ \Delta^{4}(\vert k\vert )\Delta^{4}([k])} \end{aligned}$$

$\forall k \in\mathbf{Z}^{\mathbf{N}}\backslash\{0\}$, *with a constant* $\alpha>0$ *and an approximation function* $\Delta(t)$. *Let* $0<\overline{\rho}<\rho$, $0<\overline{z}<z$. *Then*, *a symplectic change of variables* $x= y+\underline{x}$ *exists*, *so that the Hamiltonian system* (17) *can be transformed into the Hamiltonian system*

$$\begin{aligned} \frac{dy}{dt} = J \bigl[{A} + \varepsilon Q^{*} \bigr]y + \varepsilon^{2} g^{*}(t) + h^{*}(y,t) \end{aligned}$$

*such that*

$$\begin{aligned} \big|\!\big|\!\big|Q^{*}\big|\!\big|\!\big|_{z-\overline{z},\rho-\overline{\rho}}\leq |\!|\!|Q|\!|\!|_{z, \rho} + cK \frac{\Gamma(\overline{z})\Gamma(\overline{\rho})}{ \alpha} |\!|\!|{g}|\!|\!|_{z,\rho} \end{aligned}$$

*and*

$$\begin{aligned} \big|\!\big|\!\big|g^{*}\big|\!\big|\!\big|_{z-\overline{z},\rho-\overline{\rho}}\leq c \frac{ \Gamma(\overline{z})\Gamma(\overline{\rho})}{\alpha} |\!|\!|{Q}|\!|\!|_{z, \rho} |\!|\!|g|\!|\!|_{z,\rho}+ cK \biggl( \frac{\Gamma(\overline{z})\Gamma (\overline{ \rho})}{\alpha} \biggr)^{2} |\!|\!|g |\!|\!|^{2}_{z,\rho}, \end{aligned}$$

*where* $y \in B_{b_{1}}(0)$, $b_{1}= b-|\!|\!|\underline{x}|\!|\!|_{z- \overline{z},\rho-\overline{\rho}}$, *and* $\Gamma(\rho)= \sup_{t \geq0}[\Delta^{4}(t) e^{-\rho t}$.

### Proof

Consider that the equation $\frac{dx}{dt} = JA x + \varepsilon g(t)$ has solution $\underline{x}$. Using Lemma 3.4, we get

$$\begin{aligned} |\!|\!|\underline{x}|\!|\!|_{z-\overline{z},\rho-\overline{\rho}}\leq c \varepsilon \frac{\Gamma(\overline{z})\Gamma(\overline{\rho})}{ \alpha} |\!|\!|{g}|\!|\!|_{z,\rho}. \end{aligned}$$

Using the symplectic transformation $x= y+ \underline{x} $, equation (17) becomes

$$\begin{aligned} \frac{dy}{dt} = J \bigl[{A} + \varepsilon Q^{*} \bigr]y + \varepsilon^{2} g^{*}(t) + h^{*}(y,t), \end{aligned}$$

where

$$\begin{aligned}& g^{*}(t)= \frac{1}{\varepsilon^{2}}h(\underline{x},t) + \frac{1}{ \varepsilon}JQ(t) \underline{x}(t), \\& Q^{*}(t)= Q(t) + \frac{1}{\varepsilon}D_{x} h( \underline{x},t), \\& h^{*}(y,t)= h \bigl(\underline{x}(t) +y,t \bigr) - h( \underline{x},t)- JD_{x} h \bigl( \underline{x}(t),t \bigr)y. \end{aligned}$$

By Lemmas 3.4 and 3.5, we have

$$\begin{aligned} \big|\!\big|\!\big|Q^{*}\big|\!\big|\!\big|_{z-\overline{z},\rho-\overline{\rho}} \leq&|\!|\!|Q |\!|\!|_{z, \rho} + \frac{1}{\varepsilon}K |\!|\!|\underline{x}|\!|\!|_{z-\overline{z}, \rho-\overline{\rho}} \\ \leq&|\!|\!|Q|\!|\!|_{z,\rho} + cK \frac{\Gamma(\overline{z})\Gamma (\overline{ \rho})}{\alpha} |\!|\!|g|\!|\!|_{z,\rho}. \end{aligned}$$

For the estimation of $|\!|\!|g^{*}|\!|\!|_{z-\overline{z},\rho-\overline{ \rho}}$, by Lemmas 3.4 and 3.5, we have

$$\begin{aligned} \big|\!\big|\!\big|g^{*}\big|\!\big|\!\big|_{z-\overline{z},\rho-\overline{\rho}} \leq& c \frac{K}{2 \varepsilon^{2}} |\!|\!|\underline{x}|\!|\!|^{2}_{z-\overline{z},\rho-\overline{ \rho}}+ \frac{1}{\varepsilon} |\!|\!|Q|\!|\!|_{z-\overline{z},\rho-\overline{ \rho}} \\ \leq& c \frac{\Gamma(\overline{z})\Gamma(\overline{\rho})}{\alpha }|\!|\!|{Q}|\!|\!|_{z,\rho} |\!|\!|g|\!|\!|_{z,\rho}+ cK \biggl(\frac{\Gamma(\overline{z}) \Gamma(\overline{\rho})}{\alpha} \biggr)^{2} |\!|\!|g |\!|\!|^{2}_{z,\rho}. \end{aligned}$$

 □

### Lemma 3.8

*Let* $\{\delta_{m}\}$ *be a sequence of real positive numbers such that*

$$\begin{aligned} \delta_{m+1}\leq \bigl(\overline{\gamma}r^{m} \bigr)^{\overline{\gamma}r^{m}} \delta^{2}_{m} \end{aligned}$$

*for all* $m\geq0$, *where* $\overline{\gamma}>0$ *and* $1< r <2$. *Then*

$$\begin{aligned} \delta_{m}\leq \bigl[ \bigl(\overline{\gamma}r^{\frac{r}{2-r}} \bigr)^{\frac{\overline{ \gamma}}{2-r}}{\delta_{0}} \bigr]^{2m}. \end{aligned}$$

*For the proof*, *see* [5].

### Lemma 3.9

*Let* $f:[-\varepsilon,\varepsilon]\rightarrow \mathbf{C}$ *be Lipschitz from above* (*with constant* $C_{f}$) *and from below* (*with constant* $c_{f}$), *that is*,

$$\begin{aligned} \bigl\vert f(u)-f(v) \bigr\vert \leq C_{f}\vert u-v\vert , \qquad \bigl\vert f(u)-f(v) \bigr\vert \geq c_{f}\vert u-v \vert . \end{aligned}$$

*Let* $g:[-\varepsilon,\varepsilon]\rightarrow\mathbf{C}$ *be another Lipschitz from above* (*with constant* $\delta< c_{f}$), *that is*,

$$\begin{aligned} \bigl\vert g(u)-g(v) \bigr\vert \leq\delta \vert u-v\vert . \end{aligned}$$

*Then* $h=f+g$ *is a Lipschitz function from above* (*with constant* $C_{f}+\delta$) *and from below* (*with constant* $c_{f}-\delta$)

$$\begin{aligned} \bigl\vert h(u)-h(v) \bigr\vert \leq(C_{f}+\delta)\vert u-v \vert , \qquad \bigl\vert h(u)-h(v) \bigr\vert \geq(c_{f}- \delta) \vert u-v\vert . \end{aligned}$$

The proof is elementary.

### Lemma 3.10

*Suppose that* $B_{1}$ *has the eigenvalues* $\lambda^{1}_{1},\lambda^{1}_{2}, \ldots, \lambda^{1}_{2N}$ *which satisfy* $|\lambda^{1}_{s}|>2\nu$, $|\lambda^{1}_{s}-\lambda^{1}_{j}|>2 \nu$, *and* $\nu>0$ *with* $s \neq j$. *Suppose that* $A_{0}(\varepsilon)$ *satisfies* $\Vert A_{0}-B_{1}\Vert < \varsigma$ *seen in Lemma *3.3 *and* $A_{0}(\varepsilon)$ *relies on* *ε* *with constant* $L_{A_{0}}$ *in a Lipschitz way*. *Suppose that* $B(\varepsilon)$ *is the transformation that diagonalizes* $A_{0}(\varepsilon)$ (*as in Lemma *3.3). *Then there exist constants* $C_{1},C_{2} >0$ *such that*

$$\begin{aligned} \bigl\Vert B(\varepsilon_{1})-B(\varepsilon_{2})\bigr\Vert \leq C_{1} L_{A_{0}} \vert \varepsilon_{1}- \varepsilon_{2}\vert , \\ \bigl\Vert B(\varepsilon_{1})-B(\varepsilon_{2})\bigr\Vert \leq C_{1} L_{A_{0}} \vert \varepsilon_{1}- \varepsilon_{2}\vert , \\ \bigl\Vert \lambda_{j}(\varepsilon_{1})- \lambda_{j}(\varepsilon_{2})\bigr\Vert \leq C _{2} L_{A_{0}} \vert \varepsilon_{1}- \varepsilon_{2}\vert , \end{aligned}$$

*where* $\lambda_{j}(\varepsilon)$ *for all* $1 \leq j \leq2N$ *denote the eigenvalues of* $A_{0}(\varepsilon)$.

### Lemma 3.11

*Let* $\{a_{m}\}_{m}$ *be a sequence of positive real numbers which satisfy* $a_{m} \in\mathopen{]}0,1]$, $\prod^{\infty}_{m=0} a _{m}= a \in\mathopen{]}0,1]$. *Let* $\{b_{m}\}_{m}$ *be another sequence of positive real numbers satisfying* $\prod^{\infty}_{m=0} b_{m}= b < +\infty$. *Consider the new sequence* $\{\upsilon_{m}\}_{m}$ *defined by* $\upsilon_{m+1}= a_{m} \upsilon_{m}- b_{m}$. *Then the sequence* $\{\upsilon_{m}\}_{m}$ *approaches to a limit value* $\upsilon_{\infty }$ *which satisfies* $\upsilon_{\infty}\geq a \upsilon_{0}-b$.

For the proof of Lemmas 3.10 and 3.11, see [5].

## The first KAM step

Let $A_{0}=JA$, $Q_{0}(t)=JQ(t)$ be Hamiltonian matrices. First of all, for equation (1), the possible multiple eigenvalues of $A_{0}$ are changed into distinct eigenvalues and the coefficient *ε* becomes $\varepsilon^{2}$ in $Q_{0}(t)$ and $g(t)$. In the following, to simplify notations, $c >0$ denotes the different constants. Then the Hamiltonian system (1) can be rewritten as follows:

18 $$\begin{aligned} \frac{dx}{dt} = \bigl[A_{0}+\varepsilon{Q_{0}}(t) \bigr]x + \varepsilon g(t) + h(x,t), \end{aligned}$$

where $x \in B_{b}(0)$, $Q_{0}$ and *g* are analytic almost-periodic on $D_{\rho}$, and *h* is analytic almost-periodic on $\Delta_{b,\rho}$ with spatial structure $(\tau, [\cdot])$. By using the symplectic transformation $x= \underline{x}_{0} +y$, where $\underline{x}_{0}$ satisfies $\frac{d\underline{x}_{0}}{dt}= A_{0}\underline{x}_{0} + \varepsilon g(t)$ on $D_{\rho-\overline{\rho}}$, then system (18) becomes

19 $$\begin{aligned} \frac{dy}{dt} = \bigl[{A_{0}} + \varepsilon Q^{*}(t) \bigr]y + \varepsilon^{2} g ^{*}(t) + h^{*}(y,t), \end{aligned}$$

where

$$\begin{aligned}& Q^{*}= Q_{0}(t) + \frac{1}{\varepsilon}D_{x} h( \underline{x}_{0},t), \\& g^{*}(t)= \frac{1}{\varepsilon^{2}}h(\underline{x}_{0},t) + \frac{1}{ \varepsilon}Q_{0}(t) \underline{x}_{0}(t), \\& h^{*}= h(\underline{x}_{0} +y,t) - h(\underline{x}_{0},t)- JD_{x} h( \underline{x}_{0},t)y. \end{aligned}$$

By using equation (8) and Lemma 3.4, we have

20 $$\begin{aligned} |\!|\!|\underline{x}_{0}|\!|\!|_{z-\overline{z},\rho-\overline{\rho}}\leq c \varepsilon\frac{\Gamma(\overline{z})\Gamma(\overline{\rho})}{ \alpha_{0}}|\!|\!|g|\!|\!|_{z,\rho}. \end{aligned}$$

By defining the average of $Q^{*}(t)$ as $\overline{Q}^{*}$, equation (19) can be rewritten as follows:

21 $$\begin{aligned} \frac{dy}{dt} = \bigl[A_{1} + \varepsilon \widetilde{Q}(t) \bigr]y + \varepsilon ^{2} \widetilde{g}(t) + \widetilde{h}(y,t), \end{aligned}$$

where $A_{1}= A_{0} + \varepsilon\overline{Q}^{*}$, $Q^{*}(t)= \widetilde{Q}(t) +\overline{Q}^{*}$, $\widetilde{g}=g^{*}$, and $\widetilde{h}=h^{*}$. By using the conditions of Theorem 2.1, $A_{1}$ has 2*N* different eigenvalues $\lambda_{1},\lambda_{2}, \ldots, \lambda_{2N}$ which satisfy $|\lambda^{1}_{k_{1}}(\varepsilon )-\lambda^{1}_{k_{2}}(\varepsilon)|\geq2\eta\varepsilon>0$, $k_{1} \neq k_{2}$. Now, using the symplectic change of variables $y= e^{\varepsilon{P}_{0}(t)}{x}_{1}$, system (21) is transformed into the system

22 $$\begin{aligned} \frac{d{x}_{1}}{dt} =& \biggl[{e^{-\varepsilon{P}_{0}}}({A}_{1}+ \varepsilon \widetilde{Q}-\varepsilon\dot{P_{0}}){e^{\varepsilon{P}_{0}}} + {e^{-\varepsilon{P}_{0}}} \biggl(\varepsilon\dot{P_{0}}e^{\varepsilon P _{0}(t)}- \frac{d}{dt}e^{\varepsilon{P}_{0}(t)} \biggr) \biggr]{x}_{1} \\ &{}+{e^{-\varepsilon{P}_{0}}}\varepsilon^{2} \widetilde{g}(t)+{e^{- \varepsilon{P}_{0}}} \widetilde{h} \bigl(e^{\varepsilon{P}_{0}(t)}{x}_{1},t \bigr), \end{aligned}$$

where $x \in B_{b_{1}}(0)$. By series expansion, we can denote

$$\begin{aligned} e^{\varepsilon{P}_{0}}= I+ \varepsilon{P}_{0}+ {W}, \qquad e^{-\varepsilon{P}_{0}}= I- \varepsilon{P}_{0}+\widetilde{W}, \end{aligned}$$

where

$$\begin{aligned}& {W}= \frac{(\varepsilon{P}_{0})^{2}}{2!}+ \frac{(\varepsilon{P}_{0})^{3}}{3!}+\cdots, \\& \widetilde{W}= \frac{(\varepsilon{P}_{0})^{2}}{2!}-\frac{(\varepsilon {P}_{0})^{3}}{3!}+\cdots. \end{aligned}$$

Then the Hamiltonian system (22) can be rewritten as follows:

23 $$\begin{aligned} \frac{d{x}_{1}}{dt} =& \bigl(A_{1}+\varepsilon \widetilde{Q}-\varepsilon \dot{P_{0}}+\varepsilon A_{1} P_{0}-\varepsilon P_{0} A_{1} + \varepsilon^{2} Q_{1} \bigr) x_{1} \\ & {}+{e^{-\varepsilon{P}_{0}}}\varepsilon^{2} \widetilde{g}(t)+{e ^{-\varepsilon{P}_{0}}}\widetilde{h} \bigl(e^{\varepsilon{P}_{0}(t)}{x} _{1},t \bigr), \end{aligned}$$

where

$$\begin{aligned} {Q}_{1} =&-{P}_{0}(\widetilde{Q}-\dot{P_{0}})+ (\widetilde{Q}- \dot{P_{0}}){P}_{0} -{P}_{0}({A}_{1}+ \varepsilon\widetilde{Q}-\varepsilon \dot{P_{0}}){P}_{0} \\ &{}+({I}- \epsilon{P}_{0}) ({A}_{1}+\varepsilon \widetilde {Q}-\varepsilon \dot{P_{0}})\frac{W}{\varepsilon^{2}} + \frac{\widetilde{W}}{ \varepsilon^{2}}({A}_{1}+\varepsilon\widetilde{Q}-\varepsilon \dot{P_{0}})e^{\varepsilon{P}_{0}} \\ &{}+\frac{1}{\varepsilon^{2}}{e^{-\varepsilon{P}_{0}}} \biggl(\varepsilon \dot{{P}_{0}}e^{\varepsilon{P}_{0}(t)}-\frac{d}{dt}e^{\varepsilon {P}_{0}(t)} \biggr)). \end{aligned}$$

We would like to have that

$$\begin{aligned} \widetilde{Q}-\dot{P_{0}}+{A}_{1}{P}_{0}-{P}_{0}{A}_{1}=0, \end{aligned}$$

which is equivalent to

24 $$\begin{aligned} \dot{P}_{0} = {A}_{1}{P}_{0} - {P}_{0}{A}_{1} + \widetilde{Q}. \end{aligned}$$

Since $A_{0}$, $Q^{*}(t)$, and $\overline{Q}^{*}$ are Hamiltonian matrices, therefore ${A}_{1}={A_{0}}+\varepsilon\overline{Q}^{*}$ and $\widetilde{Q}(t)= Q^{*}(t)-\overline{Q}^{*}$ are also Hamiltonian matrices. Using Lemma 3.6, if

25 $$\begin{aligned} \bigl\vert \lambda^{1}_{s}- \lambda^{1}_{j}-\sqrt{-1}\langle k,\omega\rangle \bigr\vert \geq\frac{\alpha_{1}}{\Delta^{4} (\vert k\vert )\Delta^{4}([k])},\quad s\neq j, 0 \leq s,j \leq{2N} \end{aligned}$$

for all $k\in\mathbf{Z}^{\mathbf{N}}\backslash\{0\}$, $\alpha _{1}=\frac{ \alpha_{0}}{2}$, then for equation (24), a unique almost-periodic Hamiltonian matrix ${P}_{0}=\sum{P}_{0\Lambda}$ exists with the same spatial structure $(\tau,[\cdot])$ and the same frequencies as $\widetilde{Q}(t)$ on a smaller domain $D_{\rho-\overline{\rho}}$, which satisfies $\overline{P}_{0}=0$ and

26 $$\begin{aligned} |\!|\!|{P}_{0}|\!|\!|_{z-\overline{z},\rho-\overline{\rho}}\leq c \frac{ \Gamma(\overline{z})\Gamma(\overline{\rho})}{\alpha_{0}}\big|\!\big|\!\big|{Q}^{*}\big|\!\big|\!\big|_{z,\rho}. \end{aligned}$$

Therefore, by equation (24), the Hamiltonian system (23) can be rewritten as follows:

27 $$\begin{aligned} \frac{d{x}_{1}}{dt} = \bigl[{A}_{1}+ \varepsilon^{2}{Q}_{1}(t) \bigr]{x}_{1} + \varepsilon^{2} g_{1}(t) + h_{1}(x_{1},t), \end{aligned}$$

where $g_{1}(t)={e^{-\varepsilon{P}_{0}}} \widetilde{g}(t)$, $h_{1}(x_{1},t)={e^{-\varepsilon{P}_{0}}}\widetilde{h}(e^{\varepsilon {P}_{0}(t)}{x}_{1},t)$, and

$$\begin{aligned} {Q}_{1} =&-{P}_{0}({P}_{0} {A}_{1}- {A}_{1}{P}_{0})+ ({P}_{0} {A}_{1}- {A}_{1}{P}_{0}){P}_{0} -{P}_{0} \bigl({A}_{1}+\varepsilon({P}_{0} {A}_{1}- {A}_{1}{P}_{0}) \bigr) {P}_{0} \\ &{}+({I}- \varepsilon{P}_{0}) \bigl({A}_{1}+ \varepsilon({P}_{0} {A}_{1}- {A}_{1}{P}_{0}) \bigr)\frac{W}{\varepsilon^{2}} \\ &{}+\frac{\widetilde{W}}{\varepsilon^{2}} \bigl({A}_{1}+\varepsilon({P} _{0} {A}_{1}- {A}_{1}{P}_{0}) \bigr)e^{\varepsilon{P}_{0}} +\frac{1}{\varepsilon^{2}}e^{-\varepsilon P_{0}} \biggl(\varepsilon \dot{{P}_{0}}e^{\varepsilon P_{0}}- \frac{d(e^{\varepsilon P_{0}})}{dt} \biggr). \end{aligned}$$

Hence, the symplectic transformation is $T_{0} {x_{1}}= \underline{x} _{0}+ e^{\varepsilon P_{0}} {x_{1}} = \varphi_{0}(t,\varepsilon)+\psi _{0}(t,\varepsilon) {x_{1}}$. If $|\!|\!|\varepsilon P_{0}|\!|\!|_{z- \overline{z}, \rho-\overline{\rho}} \leq\frac{1}{2}$, then, by equations (20) and (26), we have

28 $$\begin{aligned}& |\!|\!|\varphi_{0}|\!|\!|_{z-\overline{z},\rho-\overline{\rho}}\leq c \varepsilon\frac{\Gamma(\overline{z})\Gamma(\overline{\rho})}{ \alpha_{0}}|\!|\!|{g}|\!|\!|_{z,\rho} , \end{aligned}$$

29 $$\begin{aligned}& |\!|\!|\psi_{0}-I|\!|\!|_{z-\overline{z},\rho-\overline{\rho}}\leq c \varepsilon \frac{\Gamma(\overline{z})\Gamma(\overline{\rho})}{\alpha_{0}}\big|\!\big|\!\big|{Q}^{*}\big|\!\big|\!\big|_{z,\rho}. \end{aligned}$$

Thus, under the symplectic change of variables $x= T_{0} {x}_{1}$, the Hamiltonian system (18) becomes Hamiltonian system (27). This completes the first KAM step.

## Proof of the main result

### The proof of Theorem 2.1

Now we will consider the standard iteration step, the proof of which is almost similar to the first KAM step. In the first step, we proved that $A_{1}$ has 2*N* different eigenvalues and $\varepsilon^{2}{Q}_{1}(t)$ and $\varepsilon^{2}{g}_{1}(t)$ are smaller perturbations. Now the KAM method will be used to prove Theorem 2.1 and we will use a similar process as that in [5] and [8]. For simplification of notations, here $c>0$ denotes the different constants. For *m*th step, consider the Hamiltonian system

30 $$\begin{aligned} \frac{d{x}_{m}}{dt} = \bigl[{A}_{m}+\varepsilon ^{{2}^{m}}{Q_{m}}(t,\varepsilon) \bigr]x_{m} + \varepsilon^{{2}^{m}} g_{m}(t,\varepsilon) + h_{m}(x_{m},t, \varepsilon), \end{aligned}$$

where $m\geq1$, $x_{m} \in B_{b_{m}}(0)$, $Q_{m}$ and $g_{m}$ are analytic almost-periodic on $D_{\rho_{m}}$, and $h_{m}$ is analytic almost-periodic on $\Delta_{b_{m},\rho_{m}}$ with basic frequencies $\omega=(\omega_{1},\omega_{2},\ldots)$ and has the spatial structure $(\tau,[\cdot])$, $A_{m} \in B_{\varsigma}(A_{1})$, and $\lambda ^{m}_{s}$ are eigenvalues of $A_{m}$ with $|\lambda^{m}_{s}(\varepsilon )-\lambda^{m}_{j}(\varepsilon)|\geq\eta\varepsilon>0$, $\forall s \neq j$ and $|\lambda^{m}_{s}(\varepsilon)|\geq\eta_{3}>0$. Using the change of variables $x_{m}= \underline{x}_{m} +y$, where $\underline{x}_{m}$ satisfies $\frac{d\underline{x}_{m}}{dt}= A_{m} \underline{x}_{m} + \varepsilon^{{2}^{m}} g_{m}(t)$ on $D_{\rho _{m}-\overline{ \rho}_{m}}$, the Hamiltonian system (30) can be written as follows:

31 $$\begin{aligned} \frac{dy}{dt} = \bigl[A_{m} + \varepsilon^{{2}^{m}}Q^{*}_{m}(t) \bigr]y+ \varepsilon^{{2}^{m+1}} g^{*}_{m}(t)+h^{*}_{m}(y,t), \end{aligned}$$

where

$$\begin{aligned}& Q^{*}_{m}(t)= Q_{m}(t) + \frac{1}{\varepsilon^{{2}^{m}}}D_{x} h _{m}(\underline{x}_{m},t), \\& g^{*}_{m}(t)= \frac{1}{\varepsilon^{{2}^{m+1}}}h_{m}( \underline{x} _{m},t) + \frac{1}{\varepsilon^{{2}^{m}}}Q_{m}(t) \underline{x}_{m}(t), \\& h^{*}_{m}(y,t)= h_{m}(\underline{x}_{m} +y,t) - h_{m}( \underline{x}_{m},t)- D_{x} h_{m}(\underline{x}_{m},t)y. \end{aligned}$$

By Lemma 3.4, if

$$\begin{aligned} \bigl\vert \lambda^{m}_{s}-\sqrt{-1}\langle k,\omega \rangle \bigr\vert \geq\frac{ \alpha_{m}}{\Delta^{4}(\vert k\vert )\Delta^{4}([k])} \end{aligned}$$

$\forall k \in\mathbf{Z}^{\mathbf{N}}\backslash\{0\}$, $\alpha _{m}=\frac{ \alpha_{0}}{2^{m}}$, and Δ is an approximation function, then we have

32 $$\begin{aligned} |\!|\!|\underline{x}_{m}|\!|\!|_{z_{m}-\overline{z}_{m},\rho_{m}-\overline{ \rho}_{m}}\leq c \varepsilon^{{2}^{m}}\frac{\Gamma(\overline{z}_{m}) \Gamma(\overline{\rho}_{m})}{\alpha_{m}}|\!|\!|g_{m}|\!|\!|_{z_{m},\rho_{m}}, \end{aligned}$$

where $0<\overline{z}_{m}<z_{m}$, $0<\overline{\rho}_{m}<\rho_{m}$, and $c>0$ is a constant. By defining the average of $Q^{*}_{m}(t)$ as $\overline{Q}^{*}_{m}$, equation (31) can be rewritten as follows:

33 $$\begin{aligned} \frac{dy}{dt} = \bigl[A_{m+1} + \varepsilon^{{2}^{m}} \widetilde{Q}_{m}(t) \bigr]y + \varepsilon^{{2}^{m+1}} \widetilde{g}_{m}(t) + \widetilde{h}_{m}(y,t), \end{aligned}$$

where $A_{m+1}= A_{m} + \varepsilon\overline{Q}^{*}_{m}$, $Q^{*}_{m}(t)= \widetilde{Q}_{m}(t)+\overline{Q}^{*}_{m}$, $\widetilde{g}_{m}=g^{*} _{m}$, and $\widetilde{h}_{m}=h^{*}_{m}$.

Now consider $\lambda^{m+1}_{1},\ldots,\lambda^{m+1}_{2N}$ to be the different eigenvalues of $A_{m+1}$ which satisfy $|\lambda^{m+1}_{k _{1}}-\lambda^{m+1}_{k_{2}}|\geq2\eta\varepsilon>0$, $k_{1} \neq k _{2}$.

By applying the symplectic transformation $y= e^{\varepsilon^{{2}^{m}} {P}_{m}(t)}x_{m+1}$, system (33) is changed into

34 $$\begin{aligned} \frac{d{x}_{m+1}}{dt} =& \biggl[{e^{-\varepsilon^{{2}^{m}}{P}_{m}}} \bigl({A}_{m+1}+ \varepsilon^{{2}^{m}}\widetilde{Q}_{m} - \varepsilon^{{2}^{m}} \dot{{P}_{m}} \bigr){e^{\varepsilon^{{2}^{m}}{P}_{m}}} \\ &{}+ {e^{-\varepsilon^{{2}^{m}}{P}_{m}}} \biggl(\varepsilon^{{2}^{m}} \dot{{P}_{m}}e^{\varepsilon^{{2}^{m}}{P}_{m}(t)} -\frac{d}{dt}e^{ \varepsilon^{{2}^{m}}{P}_{m}(t)} \biggr) \biggr]{x}_{m+1} \\ &{}+{e^{-\varepsilon^{{2}^{m}}{P}_{m}}}\varepsilon^{{2}^{m+1}} \widetilde{g}_{m}(t)+{e^{-\varepsilon^{{2}^{m}}{P}_{m}}} \widetilde{h} _{m} \bigl(e^{\varepsilon^{{2}^{m}}{P}_{m}(t)} x_{m+1},t \bigr), \end{aligned}$$

where $x_{m+1} \in B_{b_{m+1}}(0)$. By series expansion, we can denote

$$\begin{aligned} e^{\varepsilon^{{2}^{m}}{P}_{m}}= I+ \varepsilon^{{2}^{m}}{P}_{m}+ W _{m},\qquad e^{-\varepsilon^{{2}^{m}}{P}_{m}}= I- \varepsilon^{{2}^{m}}{P}_{m}+ \widetilde{W}_{m}, \end{aligned}$$

where

$$\begin{aligned} W_{m}= \frac{(\varepsilon^{{2}^{m}}{P}_{m})^{2}}{2!}+\frac{( \varepsilon^{{2}^{m}}{P}_{m})^{3}}{3!}+\cdots,\qquad \widetilde{W}_{m}= \frac{(\varepsilon^{{2}^{m}}{P}_{m})^{2}}{2!}-\frac{( \varepsilon^{{2}^{m}}{P}_{m})^{3}}{3!}+\cdots. \end{aligned}$$

Then system (34) can be rewritten as follows:

35 $$\begin{aligned} \frac{d{x}_{m+1}}{dt} =& \bigl[ {A}_{m+1}+ \varepsilon^{{2}^{m}} \widetilde{Q}_{m}-\varepsilon^{{2}^{m}} \dot{{P}}_{m}+\varepsilon^{ {2}^{m}}{A_{m+1}} {P_{m}}- \varepsilon^{{2}^{m}}{P_{m}} {A_{m+1}}+ \varepsilon^{{2}^{m+1}}{Q_{m+1}(t)} \bigr] x_{m+1} \\ &{}+{e^{-\varepsilon^{{2}^{m}}{P}_{m}}}\varepsilon^{{2}^{m+1}} \widetilde{g}_{m}(t)+{e^{-\varepsilon^{{2}^{m}}{P}_{m}}} \widetilde{h} _{m} \bigl(e^{\varepsilon^{{2}^{m}}{P}_{m}(t)} x_{m+1},t \bigr), \end{aligned}$$

where

$$\begin{aligned} {Q}_{m+1}(t) =&-{P}_{m}(\widetilde{Q}_{m}- \dot{P}_{m})+ ( \widetilde{Q}_{m}-\dot{P}_{m}){P}_{m} -{P}_{m} \bigl({A}_{m+1}+ \varepsilon^{{2}^{m}}( \widetilde{Q}_{m}-\dot{P}_{m}) \bigr){P}_{m} \\ &{}+ \bigl({I}-\epsilon^{{2}^{m}}{P}_{m} \bigr) \bigl({A}_{m+1}+\varepsilon^{{2}^{m}}( \widetilde{Q}_{m}- \dot{P}_{m}) \bigr) \frac{W_{m}}{\varepsilon^{{2}^{m+1}}} \\ &{}+\frac{\widetilde{W}_{m}}{\varepsilon^{{2}^{m+1}}} \bigl({A}_{m+1}+ \varepsilon^{{2}^{m}}( \widetilde{Q}_{m}- \dot{P}_{m}) \bigr)e^{ \varepsilon^{{2}^{m}}{P}_{m}} \\ &{}+\frac{1}{\varepsilon^{{2}^{m+1}}}{e^{-\varepsilon^{{2}^{m}}{P} _{m}}} \biggl(\varepsilon^{{2}^{m}} \dot{P}_{m} e^{\varepsilon^{{2}^{m}}{P} _{m}}- \frac{d}{dt}e^{\varepsilon^{{2}^{m}}P_{m}} \biggr). \end{aligned}$$

We would like to have

$$\begin{aligned} \widetilde{Q}_{m}- \dot{P}_{m} + A_{m+1} P_{m} - P_{m} A_{m+1} =0. \end{aligned}$$

This can be rewritten as

36 $$\begin{aligned} \dot{P}_{m} = A_{m+1} P_{m} - P_{m} A_{m+1} +\widetilde{Q}_{m}. \end{aligned}$$

Since $A_{m}$, ${Q}^{*}_{m}(t)$, and $\overline{Q}^{*}_{m}$ are Hamiltonian, therefore $A_{m+1}$ and $\widetilde{Q}_{m}(t)$ are Hamiltonian.

By Lemma 3.6, if

$$\begin{aligned} \bigl\vert \lambda^{m+1}_{s} - \lambda^{m+1}_{j} - \sqrt{-1}\langle k,\omega\rangle \bigr\vert \geq\frac{\alpha_{m}}{\Delta^{4} (\vert k\vert )\Delta^{4}([k])},\quad k\in \mathbf{Z}^{\mathbf{N}}\backslash\{0\} \end{aligned}$$

and for different eigenvalues $\lambda^{m+1}_{1},\ldots,\lambda^{m+1} _{2N}$ of $A_{m+1}$ with $|\lambda^{m+1}_{s}-\lambda^{m+1}_{j}|\geq \eta\varepsilon$, $s \neq j$ $0\leq s,j \leq{2N}$, then for equation (36), there exists a unique almost-periodic matrix ${P}_{m}(t) = \sum_{\Lambda\in\tau}{P}_{m \Lambda}(t)$ on $D_{\rho_{m}-\overline{ \rho}_{m}}$ with frequencies *ω* and spatial structure $(\tau,[\cdot])$, which satisfies

37 $$\begin{aligned} |\!|\!|{P}_{m}|\!|\!|_{{z}_{m}-\overline{{z}_{m}},\rho_{m}-\overline{\rho} _{m}}\leq c \frac{\Gamma(\overline{z}_{m})\Gamma(\overline{\rho} _{m})}{\alpha_{m}}\big|\!\big|\!\big|{Q}^{*}_{m}\big|\!\big|\!\big|_{{z}_{m},\rho_{m}}. \end{aligned}$$

Then the Hamiltonian system (35) becomes

38 $$\begin{aligned} \frac{d{x}_{m+1}}{dt}= \bigl[ {A}_{m+1}+\varepsilon ^{{2}^{m+1}}{Q_{m+1}(t)} \bigr] x _{m+1}+ \varepsilon^{{2}^{m+1}}{g}_{m+1}(t)+{h}_{m+1}(x_{m+1},t), \end{aligned}$$

where $g_{m+1}(t)={e^{-\varepsilon^{{2}^{m}}P_{m}}}\widetilde{g}_{m}(t)$, $h_{m+1}(x_{m+1},t)={e^{-\varepsilon^{{2}^{m}}P_{m}}} \widetilde{h}_{m}(e^{\varepsilon^{{2}^{m}}{P}_{m}(t)}x_{m+1},t)$, and by using $\widetilde{Q}_{m}- \dot{P}_{m} = {P}_{m} A_{m+1}- A_{m+1} {P}_{m}$, we have

39 $$\begin{aligned} Q_{m+1}(t) =& -{P}_{m}({P}_{m} A_{m+1}- A_{m+1}{P}_{m}) + ({P}_{m} A _{m+1}- A_{m+1}{P}_{m}){P}_{m} \\ &{}-{P}_{m} \bigl(A_{m+1}+\varepsilon^{{2}^{m}}({P}_{m} A_{m+1}- A_{m+1} {P}_{m}) \bigr){P}_{m} \\ &{}+ \bigl({I}- \epsilon^{{2}^{m}}{P}_{m} \bigr) \bigl(A_{m+1}+\varepsilon^{{2}^{m}}( {P}_{m} A_{m+1}- A_{m+1}{P}_{m}) \bigr) \frac{W_{m}}{\varepsilon^{{2}^{m+1}}} \\ &{}+\frac{\widetilde{W}_{m}}{\varepsilon^{{2}^{m+1}}} \bigl(A_{m+1}+ \varepsilon^{{2}^{m}}({P}_{m}A_{m+1}- A_{m+1}{P}_{m}) \bigr) e^{ \varepsilon^{{2}^{m}}{P}_{m}} \\ &{}+\frac{1}{\varepsilon^{{2}^{m+1}}}{e^{-\varepsilon^{{2}^{m}}{P} _{m}}} \biggl(\varepsilon^{{2}^{m}} \dot{{P}_{m}}e^{\varepsilon^{{2}^{m}} {P}_{m}}- \frac{d}{dt}e^{\varepsilon^{{2}^{m}}{P}_{m}(t)} \biggr). \end{aligned}$$

Thus, the symplectic transformation is $T_{m} x_{m+1} = \underline{x} _{m}+ e^{\varepsilon^{{2}^{m}}{P}_{m}} x_{m+1}=\varphi_{m}(t)+\psi _{m}(t) x_{m+1}$. If $|\!|\!|\varepsilon^{{2}^{m}}P_{m}|\!|\!|_{z_{m}- \overline{z}_{m},\rho_{m}-\overline{\rho}_{m}} \leq\frac{1}{2}$, then by equations (32) and (37), we have

40 $$\begin{aligned}& |\!|\!|\varphi_{m}|\!|\!|_{{z}_{m}-\overline{z}_{m},\rho_{m}-\overline{\rho} _{m}}\leq c \varepsilon^{{2}^{m}}\frac{\Gamma(\overline{z}_{m})\Gamma (\overline{\rho}_{m})}{\alpha_{m}}|\!|\!|{g}_{m}|\!|\!|_{{z}_{m},\rho_{m}}, \end{aligned}$$

41 $$\begin{aligned}& |\!|\!|\psi_{m}-I|\!|\!|_{{z}_{m}-\overline{z}_{m},\rho_{m}-\overline{\rho} _{m}}\leq c \varepsilon^{{2}^{m}}\frac{\Gamma(\overline{z}_{m})\Gamma (\overline{\rho}_{m})}{\alpha_{m}}\big|\!\big|\!\big|{Q}^{*}_{m} \big|\!\big|\!\big|_{{z}_{m},\rho _{m}}. \end{aligned}$$

So, using the symplectic change of variables $x_{m}= T_{m} x_{m+1}$, system (30) is transformed into system (38).

### Iteration

Now we estimate the bounds of $|\!|\!|g_{m+1}|\!|\!|_{m+1}$ and $|\!|\!|Q_{m+1}|\!|\!|_{m+1}$ as $m\rightarrow\infty$. For the *m*th step, we choose

$$\begin{aligned} \alpha_{m}=\frac{\alpha_{0}}{2^{m}},\qquad {b}_{m+1}= \frac{b_{m}-|\!|\!|\underline{x}_{m}|\!|\!|_{m}}{e^{ \varepsilon^{{2}^{m}|\!|\!|P_{m}|\!|\!|_{m+1}}}},\qquad |\!|\!|\cdot |\!|\!|_{m}=|\!|\!|\cdot |\!|\!|_{z_{m},\rho_{m}}. \end{aligned}$$

Also, suppose that $\overline{z}_{\nu}\downarrow0$ and $\overline{ \rho}_{\nu}\downarrow0$ satisfy $\sum_{\nu=0}^{\infty} \overline{z}_{\nu}=\frac{1}{2}z$ and $\sum_{\nu=0}^{\infty}\overline{ \rho}_{\nu}=\frac{1}{2}\rho$. And set $z_{m} = z-\sum_{\nu=1}^{m} \overline{z}_{\nu}$, $\rho_{m}=\rho-\sum_{\nu=1}^{m}\overline{ \rho}_{\nu}$. Assume that

$$\varphi(\rho)=\inf_{\rho_{1}+\rho_{2}+\cdots< \rho}\prod_{\nu=1} ^{\infty} \bigl[\Gamma(\rho_{\nu}) \bigr]^{2^{-\nu-1}}, $$

then

$$\varphi \biggl(\frac{1}{2}z \biggr)=\prod_{\nu=1}^{\infty} \bigl[\Gamma(\overline{z} _{\nu}) \bigr]^{2^{-\nu-1}} $$

and

$$\varphi \biggl(\frac{1}{2}\rho \biggr)=\prod _{\nu=1}^{\infty} \bigl[\Gamma(\overline{ \rho}_{\nu}) \bigr]^{2^{-\nu-1}}. $$

If $|\!|\!|\varepsilon^{{2}^{m}}P_{m}|\!|\!|_{m+1} \leq\frac{1}{2}$, we have

42 $$\begin{aligned} {b}_{m+1}\geq\frac{b_{m}-|\!|\!|\underline{x}_{m}|\!|\!|_{m}}{1+2 \varepsilon^{{2}^{m}}|\!|\!|P_{m}|\!|\!|_{m+1}}. \end{aligned}$$

By using Lemma 3.11, equation (42), and for sufficiently small *ε*, we obtain $b_{\infty}= \lim_{m\rightarrow\infty} b _{m} \geq\sigma$ with constant $\sigma>0$. By Lemma 3.7, we obtain

43 $$\begin{aligned} \big|\!\big|\!\big|{Q}^{*}_{m}\big|\!\big|\!\big|_{m+1} \leq |\!|\!|Q_{m}|\!|\!|_{m}+ c K_{m}\frac{\Gamma( \overline{z}_{m+1})\Gamma(\overline{\rho}_{m+1})}{\alpha_{m}} |\!|\!|g_{m}|\!|\!|_{m}. \end{aligned}$$

Therefore, by equations (37) and (43), we have

44 $$\begin{aligned} |\!|\!|P_{m}|\!|\!|_{m+1}\leq c K_{m} \frac{(\Gamma(\overline{z}_{m+1})\Gamma (\overline{\rho}_{m+1}))^{2}}{\alpha_{m} \alpha_{m+1}} \bigl(|\!|\!|Q_{m}|\!|\!|_{m}+ |\!|\!|g_{m}|\!|\!|_{m} \bigr). \end{aligned}$$

Set

$$\begin{aligned}& c_{1}=\max \biggl\{ c,\frac{8c}{\alpha} \biggr\} ,\qquad c_{m}= \bigl[ (m+1)^{2^{-(m+1)}}m ^{2^{-m}} \cdots2^{2^{-2}}\cdot1^{2^{-1}} \bigr] ^{2} \\& \Phi_{m}(z)=\prod_{\nu=1}^{m+1} \bigl[ \Gamma(\overline{z}_{\nu }) \bigr] ^{2^{-\nu}},\qquad \Phi_{m}(\rho)=\prod_{\nu=1}^{m+1} \bigl[ \Gamma (\overline{\rho}_{\nu}) \bigr] ^{2^{-\nu}}. \end{aligned}$$

From [9], $c_{m}$, $\Phi_{m}(z)$, $\Phi_{m}(\rho)$ are all convergent when *m* goes to infinity. Consider

$$\begin{aligned}& \mathcal{M}_{1} = \max \Bigl\{ 1,\sup_{m} \bigl(c_{1}c_{m}\Phi_{m}(z)\Phi _{m}( \rho) \bigr) \Bigr\} \big|\!\big|\!\big|{Q}(t)\big|\!\big|\!\big|_{z,\rho}, \\& \mathcal{M}_{2}= \max \Bigl\{ 1,\sup_{m} \bigl(c_{1}c_{m}\Phi_{m}(z)\Phi_{m}( \rho) \bigr) \Bigr\} \big|\!\big|\!\big|{g}(t)\big|\!\big|\!\big|_{z,\rho}, \end{aligned}$$

and set

$$\mathcal{M}= \max\{\mathcal{M}_{1},\mathcal{M}_{2}\}. $$

Firstly, we calculate $|\!|\!|g_{m+1}|\!|\!|_{m+1}$. By Lemma 3.7, we have

45 $$\begin{aligned}& |\!|\!|{g}_{m+1}|\!|\!|_{m+1} \\& \quad \leq c \frac{\Gamma(\overline{z}_{m+1})\Gamma (\overline{ \rho}_{m+1})}{\alpha_{m}} \biggl(|\!|\!|Q_{m}|\!|\!|_{m}+ c K_{m}\frac{\Gamma( \overline{z}_{m+1})\Gamma(\overline{\rho}_{m+1})}{\alpha_{m}}|\!|\!|g_{m}|\!|\!|_{m} \biggr) |\!|\!|g_{m}|\!|\!|_{m}. \end{aligned}$$

Now we estimate $|\!|\!|Q_{m+1}|\!|\!|_{m+1}$.

If $|\!|\!|\varepsilon^{{2}^{m}}P_{m}|\!|\!|_{m+1}\leq\frac{1}{2}$, we have

$$\begin{aligned} \big|\!\big|\!\big|e^{\pm{\varepsilon^{{2}^{m}}P_{m}}}\big|\!\big|\!\big|_{m+1}\leq1+\big|\!\big|\!\big|\varepsilon^{{2}^{m}}P_{m}\big|\!\big|\!\big|+ \frac{|\!|\!|\varepsilon^{{2}^{m}}P_{m}|\!|\!|^{2}}{2!}+\cdots \leq2. \end{aligned}$$

Moreover, we have that

$$\begin{aligned}& \varepsilon^{{2}^{m}}\dot{{P}_{m}}e^{\varepsilon^{{2}^{m}}{P}_{m}}- \frac{d}{dt}e^{\varepsilon^{{2}^{m}}{P}_{m}(t)} \\& \quad = \biggl(\varepsilon^{ {2}^{m}} \dot{{P}_{m}}e^{\varepsilon^{{2}^{m}}{P}_{m}}+ \varepsilon^{{2}^{m}} \dot{{P}_{m}} \frac{(\varepsilon^{{2}^{m}}P_{m})^{2}}{2!} +\varepsilon^{{2}^{m}} \dot{{P}_{m}}\frac{(\varepsilon^{{2}^{m}}P_{m})^{3}}{3!}+\cdots \biggr) \\& \qquad {}-\frac{d(\frac{(\varepsilon^{{2}^{m}}P_{m})^{2}}{2!}+\frac{( \varepsilon^{{2}^{m}}P_{m})^{3}}{3!}+\cdots)}{dt}. \end{aligned}$$

If $|\!|\!|\varepsilon^{{2}^{m}}P_{m}|\!|\!|_{m+1}\leq\frac{1}{2}$, by

$$\begin{aligned} \bigg|\!\bigg|\!\bigg|\frac{d}{dt} \bigl(\varepsilon^{{2}^{m}}P_{m} \bigr)^{n} \bigg|\!\bigg|\!\bigg|\leq n \big|\!\big|\!\big|\varepsilon^{{2}^{m}} \dot{{P}_{m}}\big|\!\big|\!\big|\big|\!\big|\!\big|\varepsilon^{{2}^{m}}P_{m} \big|\!\big|\!\big|^{n-1},\quad \text{for } n\in\mathbf{Z}^{+}, \end{aligned}$$

we obtain that

$$\begin{aligned} \bigg|\!\bigg|\!\bigg|\varepsilon^{{2}^{m}}\dot{{P}_{m}}e^{\varepsilon^{{2}^{m}}{P}_{m}}- \frac{d}{dt}e^{\varepsilon^{{2}^{m}}{P}_{m}(t)} \bigg|\!\bigg|\!\bigg|_{m+1} \leq4\big|\!\big|\!\big|\varepsilon^{{2}^{m}}\dot{{P}_{m}}\big|\!\big|\!\big|_{m+1} \big|\!\big|\!\big|\varepsilon^{{2}^{m}}P_{m}\big|\!\big|\!\big|_{m+1}. \end{aligned}$$

By equations (36) and (43), we have

46 $$\begin{aligned}& \bigg|\!\bigg|\!\bigg|\varepsilon^{{2}^{m}}\dot{{P}_{m}}e^{\varepsilon^{{2}^{m}}{P}_{m}}- \frac{d}{dt}e^{\varepsilon^{{2}^{m}}{P}_{m}(t)} \bigg|\!\bigg|\!\bigg|_{m+1} \\& \quad \leq c \varepsilon^{{2}^{m+1}} \biggl(|\!|\!|P_{m}|\!|\!|^{2}_{m+1}+|\!|\!|P_{m}|\!|\!|_{m+1} |\!|\!|Q_{m}|\!|\!|_{m} \\& \qquad {}+|\!|\!|P_{m}|\!|\!|_{m+1}cK_{m} \frac{\Gamma(\overline{z}_{m+1})\Gamma (\overline{ \rho}_{m+1})}{\alpha_{m}}|\!|\!|g_{m}|\!|\!|_{m} \biggr). \end{aligned}$$

Also, if $|\!|\!|\varepsilon^{{2}^{m}}P_{m}|\!|\!|_{m+1}\leq\frac{1}{2}$, we have

47 $$\begin{aligned} |\!|\!|W_{m}|\!|\!|_{m+1}, |\!|\!|\widetilde{W}_{m}|\!|\!|_{m+1}\leq2\big|\!\big|\!\big|\varepsilon^{{2}^{m}}P_{m}\big|\!\big|\!\big|^{2}_{m+1}. \end{aligned}$$

So, by equations (40),(46), and (47), we have

$$\begin{aligned}& |\!|\!|Q_{m+1}|\!|\!|_{m+1} \\& \quad \leq c K_{m}\frac{\Gamma(\overline{z}_{m+1}) \Gamma(\overline{\rho}_{m+1})}{\alpha_{m}} \bigl(|\!|\!|P_{m} |\!|\!|^{2}_{m+1}+ |\!|\!|P_{m}|\!|\!|_{m+1}|\!|\!|Q_{m}|\!|\!|_{m}+|\!|\!|P_{m}|\!|\!|_{m+1} |\!|\!|g_{m}|\!|\!|_{m} \bigr). \end{aligned}$$

Then, by using equation (43), the above equation can be written as follows:

48 $$\begin{aligned} |\!|\!|Q_{m+1}|\!|\!|_{m+1}\leq c K^{3}_{m}\frac{(\Gamma(\overline{z}_{m+1}) \Gamma(\overline{\rho}_{m+1}))^{5}}{\alpha^{3}_{m} \alpha^{2}_{m+1}} \bigl(|\!|\!|Q_{m} |\!|\!|^{2}_{m}+|\!|\!|g_{m}|\!|\!|^{2}_{m}+ |\!|\!|Q_{m}|\!|\!|_{m}|\!|\!|g_{m}|\!|\!|_{m} \bigr). \end{aligned}$$

For the estimate of $|\!|\!|Q_{m}|\!|\!|_{m}$ and $|\!|\!|g_{m}|\!|\!|_{m}$, we define

$$\begin{aligned} \delta_{m}= \max \bigl\{ |\!|\!|Q_{m}|\!|\!|_{m}, |\!|\!|g_{m}|\!|\!|_{m}\bigr\} . \end{aligned}$$

By, equations (45) and (48), we have that

49 $$\begin{aligned} \delta_{m+1} \leq c K^{3}_{m} \frac{(\Gamma(\overline{z}_{m+1})\Gamma (\overline{\rho}_{m+1}))^{5}}{\alpha^{3}_{m} \alpha^{2}_{m+1}}\delta ^{2}_{m}, \end{aligned}$$

here $c>0$ is a constant depending on *ρ*, *z*, and $\mathcal{M}$.

If $|\!|\!|\varepsilon^{{2}^{l}}P_{l}|\!|\!|\leq\frac{1}{4}$ for $l=0,1,2, \ldots, m-1$, then we have $K_{m}\leq(\frac{9}{2})^{m}K$ (as given below). It is immediate to check that we can find $\overline{\gamma}>0$ such that $\frac{cK^{3}_{m}}{\alpha^{3}_{m} \alpha^{2}_{m+1}}(\Gamma (\overline{z}_{m+1})\Gamma(\overline{\rho}_{m+1}))^{5} \leq(\overline{ \gamma}r^{m})^{\overline{\gamma}r^{m}}$, where $1< r<2$.

Using Lemma 3.8, we have $\delta_{m} \leq M^{{2}^{m}}$, where $M=(\overline{\gamma}r^{\frac{r}{2-r}})^{ \frac{\overline{\gamma}}{2-r}}\delta_{0}$. Thus, we have

50 $$\begin{aligned} |\!|\!|Q_{m}|\!|\!|_{m} \leq M^{{2}^{m}}, \qquad |\!|\!|g_{m}|\!|\!|_{m} \leq M^{{2}^{m}}. \end{aligned}$$

If $0<\varepsilon M<1$, then we obtain

51 $$\begin{aligned} \lim_{m\rightarrow\infty}\varepsilon^{{2}^{m}}|\!|\!|Q_{m}|\!|\!|_{m} =0, \qquad \lim_{m\rightarrow\infty} \varepsilon^{{2}^{m}}|\!|\!|g_{m}|\!|\!|_{m} =0. \end{aligned}$$

Now, we bound $\Vert A_{m}\Vert $. By (43), we have

52 $$\begin{aligned} \Vert A_{m+1}-A_{m}\Vert \leq \big|\!\big|\!\big|\varepsilon^{{2}^{m}}Q^{*}_{m}\big|\!\big|\!\big|_{m+1} \leq\varepsilon^{{2}^{m}} \biggl(|\!|\!|Q_{m} |\!|\!|_{m} + c K_{m}\frac{\Gamma( \overline{z}_{m+1})\Gamma(\overline{\rho}_{m+1})}{\alpha_{m}}|\!|\!|g_{m}|\!|\!|_{m} \biggr). \end{aligned}$$

By $c K_{m}\frac{\Gamma(\overline{z}_{m+1})\Gamma(\overline{\rho} _{m+1})}{\alpha_{m}}\leq M^{{2}^{m}}_{1}$ with suitable constant $M_{1} >0 $ and (50), if $\varepsilon M_{1} M<1$, then as $m\rightarrow\infty$, $\Vert A_{m+1}-A_{m}\Vert \rightarrow0$. Hence, $A_{m}$ is convergent, as $m\rightarrow\infty$. Suppose

53 $$\begin{aligned} \lim_{m\rightarrow\infty} A_{m}= A_{*}. \end{aligned}$$

Also, by (40), (41), (43), and (50), we have

54 $$\begin{aligned} \lim_{m\rightarrow\infty} |\!|\!|\varphi_{m}|\!|\!|_{m+1}=0, \qquad \lim_{m\rightarrow\infty} |\!|\!|\psi_{m}-I|\!|\!|_{m+1}=0. \end{aligned}$$

Let $D_{\frac{1}{2}z,\Delta_{b,\frac{1}{2}\rho}}=\bigcap^{\infty}_{m=0} D_{z_{m},\Delta_{b_{m},\rho_{m}}}$. By equations (28), (29), (40), and (41), and by the conditions of Theorem 2.1, suppose $T^{m}=T_{0} \circ T_{1} \circ\cdots\circ T_{m-1}$. And the convergence of $T^{m}$ is easy to prove on $D_{\frac{1}{2}z,\Delta_{b,\frac{1}{2} \rho}}$ as $m \rightarrow\infty$. Let $T^{m}\rightarrow T$ on $D_{\frac{1}{2}z,\Delta_{b,\frac{1}{2}\rho}}$ as $m \rightarrow \infty$. To bound $|\!|\!|P_{m}|\!|\!|_{m+1}$. For (44), it is not difficult to choose $c K_{m}\frac{(\Gamma(\overline{z}_{m+1})\Gamma(\overline{ \rho}_{m+1}))^{2}}{\alpha_{m} \alpha_{m+1}}\leq M^{{2}^{m}}_{2}$ with suitable constant $M_{2} >0 $. So, by (50), if $\varepsilon M_{2} M<1$, we have

$$\begin{aligned} \lim_{m\rightarrow\infty}\varepsilon^{{2}^{m}} |\!|\!|P_{m} |\!|\!|_{m+1}=0. \end{aligned}$$

This allows us to have the condition $\varepsilon^{{2}^{m}} \Vert P_{m} \Vert _{m+1}\leq\frac{1}{4}$ without reducing the value of *ε* at each step. Now we will show that $K_{m}$ is convergent for $m\rightarrow\infty$. By (38), we have

55 $$\begin{aligned} |\!|\!|D_{x_{m+1}x_{m+1}} h_{m+1}|\!|\!|_{z_{m+1}, \Delta_{b_{m+1},\rho_{m+1}}} \leq\frac{(1+2|\!|\!|\varepsilon^{{2}^{m}}P_{m}|\!|\!|_{m+1})^{2}}{1-2|\!|\!|\varepsilon^{{2}^{m}}P_{m}|\!|\!|_{m+1}}K_{m}. \end{aligned}$$

So, if $\varepsilon^{{2}^{m}} |\!|\!|P_{m}|\!|\!|_{m+1}\leq\frac{1}{4}$, we already have $K_{m}\leq(\frac{9}{2})^{m}K$. By the inequality $\frac{1}{1-x} \leq1+2x$, if $\frac{1}{4}\leq x \leq\frac{1}{2}$ and (55), we obtain

56 $$\begin{aligned} K_{m+1}\leq \bigl(1+4\varepsilon^{{2}^{m}}|\!|\!|P_{m}|\!|\!|_{m+1} \bigr)^{3}K_{m}. \end{aligned}$$

Using the convergent bound of $|\!|\!|P_{m}|\!|\!|_{m+1}$ and since $K_{m}\leq(\frac{9}{2})^{m}K$, then by (56) it is easy to obtain that the value of $K_{m}$ is convergent for $m \rightarrow\infty$. Suppose

57 $$\begin{aligned} \lim_{m\rightarrow\infty} K_{m} = K_{*}. \end{aligned}$$

Thus, $\lim_{m\rightarrow\infty} h_{m}= h_{*}(y,t)=O(y^{2})$ as $y\rightarrow0$.

Hence, using the symplectic change of variables $x = Ty= \varphi(t)+ \psi(t)y$ on $D_{\frac{1}{2}z,\Delta_{b,\frac{1}{2}\rho}}$, system (1) is changed into the Hamiltonian system (10).

### Measure estimate

Now, for small enough $\varepsilon_{0}$, we will prove that the non-resonant conditions

$$\begin{aligned} \bigl\vert \lambda^{m}_{s}-\sqrt{-1}\langle k,\omega \rangle \bigr\vert \geq\frac{ \alpha_{m}}{\Delta^{4}(\vert k\vert )\Delta^{4}([k])} \end{aligned}$$

and

$$\begin{aligned} \bigl\vert \lambda^{m}_{s} - \lambda^{m}_{j} - \sqrt{-1}\langle k,\omega\rangle \bigr\vert \geq\frac{\alpha_{m}}{\Delta^{4} (\vert k\vert )\Delta^{4}([k])} \end{aligned}$$

for most $\varepsilon\in(0,\varepsilon_{0})$ hold, where $1\leq s,j \leq2N$, $m=0,1,2, \ldots$ , $k\in\mathbf{Z}^{\mathbf{N}}\backslash \{0\}$, and $\Delta(t)$ is an approximation function.

Consider the Lipschitz constants from below and above of $f(\varepsilon )$ are $l(f(\varepsilon))$ and $L(f(\varepsilon))$, respectively. For any loss of generality, we can suppose that $\lambda^{m}_{s}-\lambda ^{m}_{j}$ are pure imaginary numbers. So, we suppose

58 $$\begin{aligned} \bigl\vert \lambda^{m}_{s} - \lambda^{m}_{j} - \sqrt{-1}\langle k,\omega\rangle \bigr\vert \geq\frac{\alpha_{m}}{\Delta^{4} (\vert k\vert )\Delta^{4}([k])}, \end{aligned}$$

where $\lambda^{m}_{s}(\varepsilon)-\lambda^{m}_{j}(\varepsilon)$ satisfies $l(\lambda^{m}_{s}(\varepsilon)-\lambda^{m}_{j}(\varepsilon ))\geq\eta_{0}>0$ for a constant $\eta_{0}$ with $s\neq j$.

#### Remark

Assume that $A_{m}(\varepsilon)$ is a Lipschitz function of *ε* and $L(A_{m})-L(A_{1}) = O(\varepsilon)$. By using Lemmas 3.9 and 3.10, and hypothesis (3) of Theorem 2.1, it is easy to prove that the eigenvalues $\lambda^{m}_{s}$ and the differences $\lambda^{m}_{s}-\lambda^{m}_{j}$ are Lipschitz from below and above if *ε* is small enough.

The proof of the above remark can be seen in [5].

By condition (2) of Theorem 2.1, for $m=0$, equation (58) holds. And, by condition (2) of Theorem 2.1, we obtain that (58) also holds for $s=j $. Let

$$\begin{aligned} f (\varepsilon)= \lambda^{m}_{s} - \lambda^{m}_{j} - \sqrt{-1}\langle k,\omega\rangle, \quad s \neq j \end{aligned}$$

and

$$\begin{aligned} O ^{k}_{sjm}= \biggl\{ \varepsilon\in(0, \varepsilon_{0}): \bigl\vert f (\varepsilon) \bigr\vert < \frac{\alpha_{m}}{\Delta^{4} (\vert k\vert )\Delta^{4}([k])} \biggr\} , \end{aligned}$$

such that system (30) converges for $\varepsilon\in(0,\varepsilon _{0})$ whenever $\varepsilon_{0}$ is small enough and

59 $$\begin{aligned} l \bigl(\lambda^{m}_{s}(\varepsilon) - \lambda^{m+1}_{j}(\varepsilon) \bigr)\vert \geq \eta_{0}. \end{aligned}$$

Since

$$\begin{aligned} \bigl\vert f(\varepsilon) \bigr\vert \geq& \bigl\vert \lambda_{s} - \lambda_{j}- \sqrt{-1}\langle k,\omega\rangle \bigr\vert -2M \varepsilon \\ \geq&\frac{\alpha_{0}}{\Delta(\vert k\vert )\Delta([k])}-2M\varepsilon_{0}. \end{aligned}$$

For $|k|\leq m$, $[k]\leq m$, and if $\frac{1}{4\Delta(|k|)\Delta([k])} >\frac{M\varepsilon_{0}}{\alpha _{0}}$, then

$$\begin{aligned} \bigl\vert f(\varepsilon) \bigr\vert \geq&\frac{\alpha_{0}}{\Delta(\vert k\vert )\Delta([k])}- \frac{ \alpha_{0}}{2\Delta(m)\Delta(m)} \\ \geq&\frac{\alpha_{0}}{\Delta^{4}(\vert k\vert )\Delta^{4}([k])} \\ \geq&\frac{\alpha_{m}}{\Delta^{4}(\vert k\vert )\Delta^{4}([k])} \end{aligned}$$

and $O^{k}_{sjm}=\phi$.

Let, for $|k|\geq m$, $[k] \geq m$, and if $\frac{1}{4\Delta(|k|) \Delta([k])} < \frac{\eta\varepsilon_{0}}{\alpha_{0}}$. By equation (59), we have

$$\begin{aligned}& \operatorname{mes}\bigl(O^{k}_{sjm} \bigr) < \frac{\alpha_{m}}{\Delta^{4}(\vert k\vert )\Delta^{4}([k]) \eta_{0}}, \\& \operatorname{mes}\biggl(\bigcup_{s\neq j} \bigcup _{k\in\mathbf{Z}^{\mathbf{N}}\backslash{\{0\}}}O^{k}_{sjm} \biggr) \leq\sum _{{1\leq s,j\leq2d},{k:\vert k\vert \geq m,[k]\geq m}} \operatorname{mes}\bigl(O^{k}_{sjm} \bigr) \\& \hphantom{\operatorname{mes}\biggl(\bigcup_{s\neq j} \bigcup _{k\in\mathbf{Z}^{\mathbf{N}}\backslash{\{0\}}}O^{k}_{sjm} \biggr)}\leq\frac{cd^{2}\alpha_{m}\varepsilon_{0}}{\alpha\Delta^{3}(m)} \sum_{k\in\mathbf{Z}^{\mathbf{N}}\backslash{\{0\}}} \frac{1}{\Delta (\vert k\vert )\Delta[k]} \\& \hphantom{\operatorname{mes}\biggl(\bigcup_{s\neq j} \bigcup _{k\in\mathbf{Z}^{\mathbf{N}}\backslash{\{0\}}}O^{k}_{sjm} \biggr)}\leq\frac{c\varepsilon^{2}_{0}}{\Delta^{3}(m)}. \end{aligned}$$

Let

$$\begin{aligned} E_{m}= \biggl\{ \varepsilon\in(0,\varepsilon_{0}): \bigl\vert \lambda^{m}_{s} -\lambda^{m}_{j} - \sqrt{-1}\langle k,\omega\rangle \bigr\vert \geq\frac{ \alpha_{m}}{\Delta^{4}(\vert k\vert )\Delta^{4}([k])}, k\in \mathbf{Z}^{ \mathbf{N}}\backslash{0}, s \neq j \biggr\} . \end{aligned}$$

Then

$$(0,\varepsilon_{0})-E_{m}=\bigcup _{s\neq j} \bigcup_{k\in\mathbf{Z}^{\mathbf{N}}\backslash{\{0\}}}O^{k}_{sjm}. $$

Thus

$$\operatorname{mes}\bigl((0,\varepsilon_{0})- E_{m} \bigr)=\leq \frac{c\varepsilon^{2}_{0}}{ \Delta^{3}(m)}. $$

Let $E= \bigcap^{\infty}_{m=1} E_{m}$, then

$$\operatorname{mes}\bigl((0,\varepsilon_{0})-E_{m} \bigr) \leq c \varepsilon^{2}_{0}, $$

and

$$\lim_{\varepsilon_{0}\rightarrow0} \frac{\operatorname{mes}((0,\varepsilon_{0})-E _{m})}{\varepsilon_{0}}=0. $$

So, if $\varepsilon_{0}$ is sufficiently small, *E* is a non-empty subset of $(0,\varepsilon_{0})$. Similar to the above, for sufficiently small $\varepsilon_{0}$, $|\lambda^{m}_{s}-\sqrt{-1}\langle k, \omega\rangle|\geq \frac{\alpha_{m}}{\Delta^{4}(|k|)\Delta^{4}([k])}$ holds for most $\varepsilon\in(0,\varepsilon_{0})$. Hence,we proved that system (1) is changed into the Hamiltonian system (10).

## Conclusion

In this work, we discussed the reducibility of almost-periodic Hamiltonian systems and proved that the almost-periodic non-linear Hamiltonian system (1) is reduced to a constant coefficients Hamiltonian system with an equilibrium by means of an almost-periodic symplectic transformation. The result was proved for a sufficiently small parameter *ε* by using some non-resonant conditions, non-degeneracy conditions, the suitable hypothesis of analyticity, and KAM iterations.
